# Natural flavonoids from herbs and nutraceuticals as ferroptosis inhibitors in central nervous system diseases: current preclinical evidence and future perspectives

**DOI:** 10.3389/fphar.2025.1570069

**Published:** 2025-03-24

**Authors:** Qiuhe Li, Xiaohang Yang, Tiegang Li

**Affiliations:** ^1^ Department of Emergency Medicine, Shengjing Hospital of China Medical University, Shenyang, China; ^2^ Department of Obstetrics and Gynecology, Qilu Hospital of Shandong University, Jinan, China

**Keywords:** ferroptosis inhibitors, flavonoids, natural compounds, central nervous system diseases, molecular mechanisms

## Abstract

Flavonoids are a class of important polyphenolic compounds, renowned for their antioxidant properties. However, recent studies have uncovered an additional function of these natural flavonoids: their ability to inhibit ferroptosis. Ferroptosis is a key mechanism driving cell death in central nervous system (CNS) diseases, including both acute injuries and chronic neurodegenerative disorders, characterized by iron overload-induced lipid peroxidation and dysfunction of the antioxidant defense system. This review discusses the therapeutic potential of natural flavonoids from herbs and nutraceuticals as ferroptosis inhibitors in CNS diseases, focusing on their molecular mechanisms, summarizing findings from preclinical animal models, and providing insights for clinical translation. We specifically highlight natural flavonoids such as Baicalin, Baicalein, Chrysin, Vitexin, Galangin, Quercetin, Isoquercetin, Eriodictyol, Proanthocyanidin, (−)-epigallocatechin-3-gallate, Dihydromyricetin, Soybean Isoflavones, Calycosin, Icariside II, and Safflower Yellow, which have shown promising results in animal models of acute CNS injuries, including ischemic stroke, cerebral ischemia-reperfusion injury, intracerebral hemorrhage, subarachnoid hemorrhage, traumatic brain injury, and spinal cord injury. Among these, Baicalin and its precursor Baicalein stand out due to extensive research and favorable outcomes in acute injury models. Mechanistically, these flavonoids not only regulate the Nrf2/ARE pathway and activate GPX4/GSH-related antioxidant pathways but also modulate iron metabolism proteins, thereby alleviating iron overload and inhibiting ferroptosis. While flavonoids show promise as ferroptosis inhibitors for CNS diseases, especially in acute injury settings, further studies are needed to evaluate their efficacy, safety, pharmacokinetics, and blood-brain barrier penetration for clinical application.

## Graphical Abstract

**Figure:**
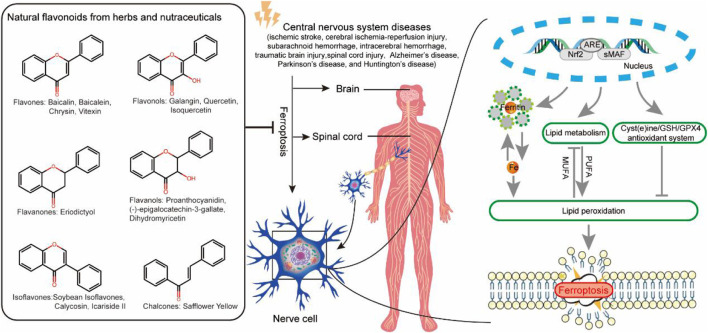
Third-party elements were sourced under CC0 (free use, no attribution) and CC BY (modifiable, commercial use with attribution). CC BY materials originate from the Freepik library (https://www.freepik.com/).

## Highlights

• Natural flavonoids ameliorate central nervous system diseases by anti-ferroptosis.
• Natural flavonoids’ ferroptosis inhibition is most studied in acute CNS injury models.
• Key mechanisms include Nrf2/ARE activation, GPX4 upregulation, and iron relief.
• Baicalin and baicalein show promise due to their iron-chelating and antioxidant effects.
• Details of representative flavonoids’ application in animal models are listed.

## 1 Introduction

Cell death is a fundamental physiological process essential for development, differentiation, and homeostasis. While regulated cell death is crucial for maintaining biological functions, its dysregulation can lead to various diseases, particularly those affecting the central nervous system (CNS) (Park et al., 2023). The CNS, comprising the brain and spinal cord, is highly susceptible to damage from trauma, infections, strokes, genetic disorders, and neurodegenerative diseases, many of which remain poorly understood and difficult to treat (Buffington and Rasband, 2011). CNS diseases, including spinal cord injury (SCI), traumatic brain injury (TBI), ischemic stroke (IS), and hemorrhagic stroke (e.g., subarachnoid hemorrhage [SAH] and intracerebral hemorrhage [ICH]), result in extensive neuronal death. Neurodegenerative diseases (NDDs), such as Alzheimer’s disease (AD), Parkinson’s disease (PD), and Huntington’s disease (HD), are also characterized by progressive neuronal loss (Waraich and Ajayan, 2024; Sanghai and Tranmer, 2023; Chi et al., 2018). Understanding the mechanisms underlying neuronal death is therefore critical to developing effective treatments for CNS diseases.

Among various forms of regulated cell death, ferroptosis has gained significant attention since its identification in 2012 (Dixon et al., 2012). Ferroptosis is an iron-dependent, lipid peroxidation-driven cell death pathway characterized by the accumulation of lipid reactive oxygen species (ROS) and disruptions in redox homeostasis, iron metabolism, and lipid regulation (Dixon and Olzmann, 2024). Excessive ferroptosis activation has been implicated in CNS diseases, making it a promising therapeutic target (Tan et al., 2021). Ferroptosis inhibitors, which primarily reduce free iron levels, scavenge ROS, and inhibit lipid peroxidation, have shown therapeutic potential in mitigating neuronal ferroptosis and slowing CNS disease progression (Zhang et al., 2024; David et al., 2022; Lane et al., 2021; David et al., 2023; Hu et al., 2021). However, their clinical application remains limited due to poor stability and biocompatibility (Nie et al., 2022).

Recently, natural compounds from herbs and nutraceuticals, particularly flavonoids, have emerged as promising ferroptosis inhibitors due to their multitarget actions, pleiotropic properties, and favorable safety profiles (Atanasov et al., 2021; Li Q. et al., 2019; Zhang S. et al., 2021; Liu L. et al., 2024; Zhao et al., 2023; Yang et al., 2023). Flavonoids, a widespread class of phenolic compounds found in plants such as fruits, vegetables, grains, and herbs, exhibit diverse biological activities, including antioxidant, anti-inflammatory, and neuroprotective effects (Testai, 2015; Dong et al., 2022). Preclinical studies have shown that flavonoids regulate ferroptosis, reducing neuronal death and slowing the progression of CNS diseases (Liu L. et al., 2024). Specific flavonoids, such as baicalein, baicalin, and quercetin, have been extensively studied in animal models for their neuroprotective effects, although their clinical efficacy remains to be established (Bellavite, 2023).

This review explores the core mechanisms of ferroptosis and its role in CNS diseases, including acute injuries (e.g., IS, CIRI, ICH, SAH, TBI, and SCI) and NDDs (e.g., AD, PD, HD). It also examines the current application of synthetic ferroptosis inhibitors in CNS diseases and their limitations. Finally, the review highlights the therapeutic potential of flavonoids as ferroptosis inhibitors, providing detailed insights into their preclinical applications, including dosage, frequency, and subclass-specific effects. Unlocking the therapeutic potential of flavonoids offers new avenues for innovative treatments targeting ferroptosis in CNS diseases.

## 2 Core mechanisms of ferroptosis

Ferroptosis is characterized by unique cellular morphological, biochemical, and genetic features. Unlike other forms of programmed cell death such as apoptosis and pyroptosis, the morphological changes in ferroptosis primarily involve mitochondrial structural alterations. These include mitochondrial shrinkage, loss of structural integrity, and increased membrane density, while the plasma membrane remains intact without swelling or rupture, and the nucleus retains its normal volume without chromatin condensation (Yu et al., 2021). Biochemically, ferroptosis is driven by the accumulation of lipid hydroperoxides (L-OOHs) resulting from lipid peroxidation of unsaturated fatty acids in cell membranes. Elevated intracellular levels of ferrous iron (Fe^2+^) or lipoxygenase (LOX) activity promote lipid peroxidation, and an imbalanced antioxidant system prevents the timely clearance of excessive L-OOHs. This redox imbalance leads to cross-linking of L-OOHs with macromolecular proteins essential for cellular functions, disruption of membrane integrity, and ultimately ferroptosis (Jin et al., 2024). Genetically, ferroptosis is associated with the aberrant expression of key genes considered its biomarkers and drivers. These include the overexpression of enzymes involved in fatty acid metabolism, such as acyl-CoA synthetase long-chain family member 4 (ACSL4), antioxidant defense enzymes like glutathione peroxidase 4 (GPX4), transcription factors such as nuclear factor E2-related factor 2 (Nrf2), and plasma membrane repair molecules like the endosomal sorting complexes required for transport III (ESCRT-III) (Chen et al., 2021a). Effectively eliminating L-OOHs or inhibiting their production is critical for preventing ferroptosis and maintaining cellular homeostasis (Du and Guo, 2022). This section will explore the mechanisms underlying L-OOH production, including iron overload, free radical chain reactions, and LOX catalysis, as well as the processes involved in their clearance.

### 2.1 Lipid peroxidation

**FIGURE 1:**
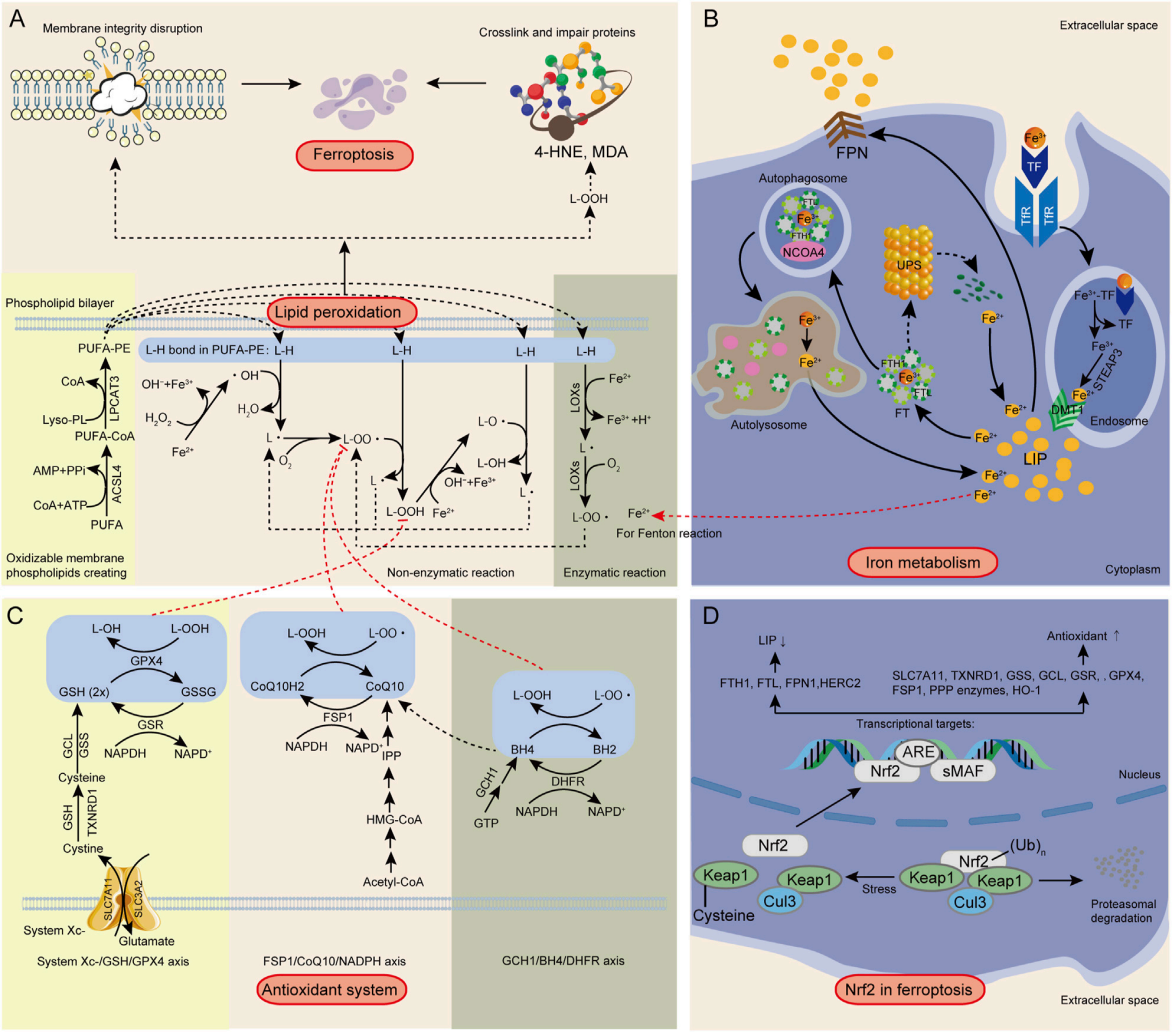
Summary of the mechanism of ferroptosis. **(A)** Lipid peroxidation. **(B)** Iron overload. **(C)** Antioxidant system dysfunction. **(D)** Nrf2/ARE pathway. Third-party elements were sourced under CC0 (free use, no attribution) and CC BY (modifiable, commercial use with attribution). CC BY materials originate from the Freepik library (https://www.freepik.com/).

In ferroptosis, lipid peroxidation refers to the oxidative degradation of polyunsaturated fatty acids (PUFAs) within phospholipids (PLs) in biological membranes by ROS, resulting in the formation of L-OOHs (Wang J. et al., 2018). PUFAs are integral components of the phospholipid bilayer, influencing lipid dynamics, protein-lipid interactions, and membrane transport properties (Dyall et al., 2022). Additionally, PUFAs serve as precursors for signaling lipids involved in physiological processes such as inflammation, synaptic plasticity, and neurodegeneration (Dyall et al., 2022). Due to their bis-allylic methylene groups, PUFAs are highly susceptible to ROS attack compared to saturated fatty acids (SFAs) and monounsaturated fatty acids (MUFAs), leading to oxidation, hydroxyl group formation, and ultimately a peroxidized state (Su et al., 2019; Ma T-L. et al., 2022). Free PUFAs do not trigger ferroptosis; only PUFAs embedded in phospholipids (PUFA-PLs) undergo peroxidation (forming PUFA-PL-OOH) and activate ferroptosis (Stockwell, 2022). While free PUFAs can be oxidized, their oxidation products are efficiently cleared by antioxidant systems and do not compromise membrane integrity. In contrast, PUFA-PL peroxidation produces L-OOHs within membrane structures, which are difficult to clear due to their molecular size. This accumulation disrupts membrane integrity, ultimately inducing ferroptosis (Figure 1A).

A recent study indicated that when PUFAs such as arachidonic acid (AA) and adrenic acid (AdA) are present, PLs, especially phosphatidylethanolamines (PEs), are more prone to oxidation (Kagan et al., 2017a). This increased susceptibility is due to the highly reactive nature of AA and AdA, which contain multiple double bonds, making them easy targets for oxidative attacks. Additionally, PEs, located in the inner layer of the cell membrane, frequently interact with Fe^2+^, which catalyzes the fenton reaction, producing highly reactive hydroxyl radicals (•OH) that readily oxidize AA and AdA. (Kagan et al., 2017a). Therefore, increasing the proportion of PEs with AA and AdA side chains in the inner layer of the biomembrane is a necessary condition to ensure lipid peroxidation during ferroptosis. Before lipid peroxidation begins, free AA and AdA are linked to CoA by ACSL4, forming AA-CoA and AdA-CoA (collectively referred to as PUFA-CoA) (Ding et al., 2023).

Lysophosphatidylcholine acyltransferase 3 (LPCAT3) then catalyzes the esterification of PUFA-CoA into PLs, particularly PEs, creating oxidizable membrane PUFA-PEs, which is more likely to undergo lethal lipid peroxidation and ferroptosis (Figure 1A; Lee et al., 2023). Thus, both ACSL4 and LPCAT3 are promising targets for combating ferroptosis and other peroxidation-related diseases. ACSL4 inhibitors, such as thiazolidinediones (TZDs), including troglitazone (TRO), pioglitazone (PIO), and rosiglitazone (ROSI), have been reported to inhibit ferroptosis in mouse embryonic fibroblasts (Tan et al., 2021). Knockdown of LPCAT3 can also confer resistance to ferroptosis in mouse lung epithelial cells and embryos (Kagan et al., 2017b). Inhibiting ACSL4 or LPCAT3 reduces the availability of substrates for lipid peroxidation, thereby preventing ferroptosis.

PUFAs, modified by ACSL4 and LPCAT3, are converted into oxidizable membrane phospholipids, generating L-OOHs through non-enzymatic and enzymatic reactions (Figure 1A). Hydroxyl radicals, among the most aggressive ROS, preferentially oxidize proteins and lipids. lipid peroxidation starts when •OH or lipid alkoxyl radicals (LO•) abstract hydrogen atoms from L-H bonds in PUFA-PEs (Collin, 2019; Angeli et al., 2017). •OH acquires hydrogen atoms to form lipid radicals (L•), also known as pentadienyl radicals), which then react with oxygen molecules to create lipid peroxyl radicals (LOO•) (Ayala et al., 2014). The close proximity of fatty acid chains in the phospholipid bilayer facilitates LOO• to abstract another hydrogen atom from adjacent PEs, forming L-OOH and a new L•, thus propagating the chain reaction and producing more L-OOHs (Yin et al., 2011). This process, known as non-enzymatic lipid peroxidation, is further fueled by Fe^2+^-dependent fenton reactions, which convert hydrogen peroxide (H_2_O_2_) to •OH and oxidize L-OOH by cleaving its O-O bond to generate LO• and hydroxide ions (OH^−^). LO• and •OH are highly reactive, initiating further lipid peroxidation chain reactions, damaging adjacent PUFA-PEs, and causing cell membrane damage and ferroptosis (Xiao et al., 2024; Gaschler and Stockwell, 2017). In enzymatic reactions, proteins containing heme or Fe-S clusters interact with specific ROS-generating enzymes such as LOXs, cytochrome P450, and cyclooxygenases (COXs) to facilitate lipid peroxidation (Ayala et al., 2014). LOXs, including various subtypes, are regulated by Fe^2+^ and can directly catalyze L-H bonds in PUFA-PEs, forming L-OO•. These radicals then abstract hydrogen atoms from neighboring PUFA-PEs, resulting in the formation of L-OOHs (Figure 1A; Jiang et al., 2021). Thus, LOXs accelerate lipid peroxidation through enzymatic pathways, contributing to the accumulation of L-OOHs and promoting ferroptosis. Karataş, et al. indicate that LOXBlock-1 (LB1) can reduce infarct volume and hemorrhage in ischemic stroke mouse models, indicating its potential to inhibit ferroptosis (Yigitkanli et al., 2013).

The exact mechanism by which L-OOHs ultimately lead to ferroptosis in cells requires further investigation. There are currently two well-established mechanisms of ferroptosis (Figure 1A). One mechanism is that the lipid peroxidation process, primarily occurring on the inner side of biological membranes, creates pores and disrupts membrane integrity. One mechanism involves lipid peroxidation on the inner side of biomembranes, which creates pores and disrupts membrane integrity (Agmon et al., 2018). Additionally, L-OOHs degrade into toxic aldehydes, such as 4-hydroxy-2-nonenal (4-HNE) and malondialdehyde (MDA), which crosslink and impair essential cellular proteins, resulting in cell death (Angeli et al., 2017; Zhong and Yin, 2015). In summary, the highly reactive primary products (L-OOHs) and secondary products (e.g., MDA and 4-HNE) generated during lipid peroxidation are toxic molecules that cause cell damage (Ayala et al., 2014).

### 2.2 Iron overload

Iron metabolism refers to the processes of absorption, transport, distribution, storage, utilization, transformation, and excretion of iron within an organism (Roemhild et al., 2021). The CNS has a high demand for blood supply, and blood components such as red blood cells and hemoglobin (Hb) eventually degrade into iron. Abnormal cellular iron metabolism leading to increased intracellular iron levels creates an environment conducive to ferroptosis (Rouault, 2013). Fe^2+^ catalyzes fenton reactions and serves as an essential component of enzymes such as LOXs (Xiao et al., 2024). Theoretically, any process that increases iron absorption, reduces iron storage, or restricts iron efflux can raise intracellular free iron levels and induce ferroptosis. Conversely, iron chelators and other agents that lower intracellular iron concentration can inhibit ferroptosis (Yan et al., 2021). Cells, particularly neurons, have developed sophisticated systems to regulate iron homeostasis through absorption, storage, and output, involving transferrin (TF)-transferrin receptor (TfR), ferritin (FT), and ferroportin (FPN) (Figure 1B; Rouault, 2013).

Firstly, Fe^3+^ binds to TF and is transported from storage sites to iron-requiring areas in the body (Figure 1B). TF carrying Fe^3+^ is recognized by TfR on the cell membrane, leading to endocytosis and formation of endosomes (Cheng et al., 2004). Within the acidic environment of endosomes, Fe^3+^ is released from TF and reduced to Fe^2+^ by metalloreductases such as STEAP3 (Zhang et al., 2012). Fe^2+^ is then transported from endosomes to the cytoplasm via divalent metal transporter 1 (DMT1) (Yanatori and Kishi, 2019). Reduced Fe^2+^forms various iron-binding complexes for physiological functions. When these complexes are saturated, excess free Fe^2+^ accumulates in the labile iron pool (LIP), accelerating fenton reactions and increasing •OH and L-O• levels, thereby promoting lipid peroxidation and inducing ferroptosis. Inhibiting iron uptake can reduce LIP levels and suppress ferroptosis (Rochette et al., 2022). For example, depleting TF from serum or using RNA interference (RNAi) to downregulate TfR significantly inhibits ferroptosis in mouse embryonic fibroblasts (Gao et al., 2015). Additionally, CD133, a cancer stem cell marker, has been found to inhibit TfR-mediated iron endocytosis, reducing intracellular iron levels and preventing ferroptosis (Yu et al., 2024; Gammella et al., 2017).

Fe^2+^ can exist in the LIP for biochemical reactions or be stored in stable proteins like FT. Intracellular Fe^2+^ can be oxidized and stored in FT, protecting cells by sequestering iron and preventing L-OOHs generation, thus inhibiting ferroptosis (Figure 1B). FT is a hollow, spherical protein shell composed of heavy (FTH1) and light (FTL) chains (Zhang N. et al., 2021). Nuclear receptor coactivator 4 (NCOA4)-related autophagosome and the ubiquitin-proteasome system (UPS) are key regulators of iron release from FT. NCOA4 primarily mediates ferritinophagy via the lysosomal pathway (Wang J. et al., 2023), while UPS regulates FT degradation under non-autophagic conditions (Li Y. et al., 2022). Inhibiting NCOA4 disrupts its binding to FTH1 and subsequent recruitment of FT complexes to lysosomes (Fang et al., 2021). Studies have shown that RNAi-mediated knockdown of NCOA4 expression significantly inhibits ferritinophagy, thereby reducing ferroptosis in mouse embryonic fibroblasts (Gao et al., 2016). Additionally, research has demonstrated that ataxia-telangiectasia-mutated (ATM) kinase can inhibit ferroptosis sensitivity by upregulating FT levels (Aki and Uemura, 2021).

Excess Fe^2+^ can also be exported out of cells via FPN, the only known vertebrate protein that actively transports iron out of cells, reducing intracellular fenton reactions and oxidative stress, ultimately inhibiting ferroptosis (Figure 1B; Ward and Kaplan, 2012). Overexpression of FPN has been shown to eliminate erastin-induced ferroptosis in ectopic endometrial stromal cells (Li et al., 2021a). In an Alzheimer’s disease mouse model, FPN gene deletion increased ferroptosis, leading to memory impairment, whereas restoring FPN improved ferroptosis and memory deficits (Bao et al., 2021). Besides being exported via the FPN pathway, elevated expression of prominin 2 (PROM2), an intracellular iron stress response protein, can facilitates the formation of ferritin-containing multivesicular bodies (MVBs) and exosomes, which effectively reduce intracellular iron levels and prevent ferroptosis by exporting iron out of the cells (Brown et al., 2019).

### 2.3 Antioxidant system

The antioxidant system is crucial for maintaining the redox balance within cells. During ferroptosis, the function of the antioxidant system is inhibited, leading to the occurrence of lipid peroxidation and ferroptosis (Wang S. et al., 2024). The primary pathways for clearing L-OOHs in ferroptosis are the cystine/glutamate antiporter system-glutathione-glutathione peroxidase 4 axis (System Xc-/GSH/GPX4 axis), the ferroptosis suppressor protein 1-coenzyme Q10-nicotinamide adenine dinucleotide phosphate axis (FSP1/CoQ10/NADPH axis), and the GTP cyclohydrolase 1-tetrahydrobiopterin-dihydrofolate reductase axis (GCH1/BH4/DHFR axis) (Figure 1C).

#### 2.3.1 System Xc-/GSH/GPX4 axis

The System Xc-/GSH/GPX4 pathway relies on GPX4 to clear cellular L-OOHs (Chen et al., 2021b). The system Xc- consists of a light chain subunit solute carrier family 7 member 11 (SLC7A11) and a heavy chain subunit solute carrier family 3 member 2 (SLC3A2) (Sato et al., 1999), linked by a disulfide bond, which transport extracellular cystine into cells in exchange for intracellular glutamate (Parker et al., 2021). The imported cystine is reduced to cysteine via the glutathione (GSH) or thioredoxin reductase 1 (TXNRD1)-dependent cystine reduction pathway (Ren et al., 2017). Cysteine is then used to synthesize GSH, a potent antioxidant, through the action of glutamate-cysteine ligase (GCL) and glutathione synthase (GSS) (Paul et al., 2018). GSH exists in reduced (GSH) and oxidized forms (GSSG). Due to the action of glutathione-S reductase (GSR), which uses electrons from NADPH/H+ to convert GSSG back to the reduced form GSH, the reduced form, which is predominant under normal conditions (Wu J. et al., 2022). GSH serves as a cofactor for GPX4, facilitating the reduction of L-OOHs to their corresponding alcohols (L-OHs), thereby preventing lipid peroxidation accumulation and ultimately inhibiting ferroptosis (Ursini and Maiorino, 2020). Inhibiting System Xc- leads to decreased GSH levels, reduced GPX4 activity, and weakened cellular antioxidant capacity, promoting ferroptosis (Li FJ. et al., 2022). Some transcription factors can regulate ferroptosis through the system Xc-/GSH/GPX4 pathway. Activating transcription factor 3 (ATF3) inhibits SLC7A11 transcription, reducing GSH synthesis and promoting ferroptosis. Conversely, activating transcription factor 4 (ATF4) activates SLC7A11 expression, enhancing GSH synthesis and inhibiting ferroptosis (Wang L. et al., 2020).

GPX4, also known as phospholipid hydroperoxide GSH-Px, is the fourth member of the selenium-containing GSH-Px family, with a molecular weight of approximately 20–21 kDa and composed of about 197 amino acids (Pei et al., 2023). GPX4 is a core regulator of ferroptosis, considered a crucial target in ferroptosis research. Its enzymatic activity is vital for cells, effectively reducing various L-OOHs and inhibiting arachidonic acid metabolism enzymes during lipid peroxidation (Xu et al., 2021). Studies show that RNAi-induced GPX4 downregulation is sufficient to induce ferroptosis, while GPX4 overexpression in HT-1080 cells confers resistance to ferroptosis, and GPX4-deficient cells are more susceptible (Yang et al., 2014). The mammalian target of rapamycin (mTOR) pathway also regulates ferroptosis. CmTOR complex 1 (mTOR1) induces cysteine-related GPX4 protein synthesis, inhibiting lipid peroxidation and protecting cells from ferroptosis (Liu Y. et al., 2021). Reduced mTOR activity decreases GPX4 protein levels and increases ROS levels, causing lipid peroxidation and ferroptosis. mTOR inhibition also reduces the expression of iron storage proteins (e.g., FTH1) and iron transport proteins (e.g., FPN), leading to iron metabolism disorders and ferroptosis (Lei et al., 2021).

#### 2.3.2 FSP1/CoQ10/NADPH and GCH1/BH4/DHFR axis

In a tumor xenograft mouse model, GPX4-ko/FSP1-ko tumor growth was inhibited, while GPX4-ko tumors grew normally, indicating that the FSP1/CoQ10/NADPH system protects cells from ferroptosis induced by GPX4 inhibition or knockout, supplementing the loss of GPX4 with an antioxidant enzyme catalytic system (Bersuker et al., 2019). FSP1 contains an N-terminal myristoylation sequence, aiding its localization to the lipid bilayer (i.e., cell membrane) and facilitating fatty acid modification (Eisenhaber et al., 2003; Borgese et al., 1996). This localization is crucial for FSP1’s biological function on the cell membrane. Anchored FSP1 uses NADPH as an electron donor to reduce CoQ10 to its reduced form (CoQ10H2) (Bebber and von Karstedt, 2023). Reduced CoQ10 is a potent antioxidant, capturing and neutralizing LOO•, preventing the propagation of radical chain reactions, and inhibiting L-OOH formation, thus preventing lipid peroxidation and ferroptosis (Bentinger et al., 2007). Screening nearly 10,000 compounds, Doll et al. identified iFSP1 as the first effective FSP1 inhibitor. iFSP1 treatment made H-1080 and mouse Pfa1 cells more susceptible to ferroptosis (Doll et al., 2019).

GCH1 plays a critical role in synthesizing BH4, essential for protecting cells from ferroptosis. GCH1 selectively protects cell membrane phospholipids from oxidative degradation, reducing lipid peroxidation and inhibiting ferroptosis (Ma T. et al., 2022). BH4, a downstream product of GCH1, directly captures and neutralizes LOO•, preventing the propagation of lipid peroxidation chain reactions (Hu et al., 2022). Additionally, BH4 is involved in CoQ10 synthesis, an important lipophilic antioxidant that captures and neutralizes lipid peroxidation radicals, protecting membrane phospholipids, especially those with two PUFA tails, from oxidative degradation (Gu et al., 2023). DHFR is crucial for regenerating BH4 from its oxidized form, maintaining BH4’s antioxidant capacity and continuous cellular protection from oxidative stress (Liang et al., 2023). Regulating GCH1, BH4, and DHFR levels effectively prevents lipid peroxidation and ferroptosis, offering new therapeutic strategies for ferroptosis-related diseases.

### 2.4 Major roles of Nfr2 in ferroptosis (via regulating downstream genes)

Nuclear factor erythroid 2-related factor 2 (NFE2L2, also known as Nrf2) is crucial for cellular antioxidant responses (Shakya et al., 2023; Yan et al., 2023). It promotes the transcription of downstream genes by binding to antioxidant response elements (ARE). Extensive research indicates that NRF2 plays a pivotal role in regulating ferroptosis due to its diverse functions in iron, lipid, and amino acid metabolism (Shakya et al., 2023; Dodson et al., 2019). Therefore, targeting Nrf2-related signaling pathways to inhibit ferroptosis has emerged as a promising therapeutic approach for combating central nervous system diseases (Song and Long, 2020). Nuclear factor erythroid 2-related factor 2 (Nrf2) is regulated by upstream mechanisms involving the Keap1 (Kelch-like ECH-associated protein 1)-Cul3 (Cullin 3)-Rbx1 (RING-box protein 1) axis. Keap1 binds to Nrf2, leading to its ubiquitination and degradation via the Cul3-Rbx1 E3 ligase complex. Under oxidative stress, Keap1 is inactivated, allowing Nrf2 to stabilize and translocate to the nucleus. In the nucleus, Nrf2 interacts with small Maf proteins (sMAF) and binds to antioxidant response elements (ARE), promoting the transcription of downstream antioxidant genes (Yan et al., 2023). This pathway is crucial for managing oxidative stress and ferroptosis.

Nrf2 plays a role in the antioxidant system by regulating the transcription of genes involved in three major pathways: the System Xc−/GSH/GPX4 axis, the FSP1/CoQ10/NADPH axis, and the GCH1/BH4/DHFR axis (Figure 1D; Yan et al., 2023). These pathways collectively contribute to cellular defense against oxidative stress and ferroptosis. In the System Xc−/GSH/GPX4 axis, Nrf2 positively regulates SLC7A11 (xCT, a subunit of System Xc−), promoting cystine import and glutamate export, thereby increasing intracellular cystine levels and facilitating GSH synthesis (Lewerenz et al., 2013). Additionally, Nrf2 regulates TXNRD1 at the transcriptional level, aiding in the reduction of cystine to cysteine (Malhotra et al., 2010). Nrf2 also regulates two key enzymes in GSH biosynthesis: GCL (composed of GCLC and GCLM subunits, catalyzing the conjugation of glutamate and cysteine) and GSS (Sasaki et al., 2002; Kwak et al., 2002; Ishii et al., 2000; Yang et al., 2005; Chan and Kwong, 2000). Nrf2 positively regulates GPX4 and GSR. GPX4, with the help of GSH, reduces peroxides, converting GSH to GSSG, while GSR, along with NADPH, reduces GSSG back to GSH (Wu et al., 2019; Amaral et al., 2019). This regulation enhances cellular antioxidant capacity and inhibits ferroptosis. In the FSP1/CoQ10/NADPH axis, Nrf2 targets and positively regulates FSP1, a lipophilic antioxidant. This regulation enhances the production of reduced CoQ10, which neutralizes L-OO•, thereby preventing lipid peroxidation and ferroptosis (Yan et al., 2023). Additionally, NADPH plays a crucial role in the antioxidant systems involving GPX4, FSP1, and DHFR, primarily as an electron donor in reduction reactions (Doll et al., 2019; Mandal et al., 2010; Soula et al., 2020). NADPH is generated through several pathways, such as the pentose phosphate pathway (PPP), NADK-catalyzed NADH phosphorylation, and IDH-catalyzed conversion of isocitrate to α-KG. Nrf2 directly regulates the transcription of various PPP enzymes, including glucose-6-phosphate dehydrogenase (G6PD) and other oxidative PPP enzymes, promoting NADPH production (Mitsuishi et al., 2012). This regulation enhances the antioxidant system and inhibits ferroptosis. Heme Oxygenase-1 (HO-1) plays a crucial role in the antioxidant system by converting heme into biliverdin, which is then reduced to bilirubin, a potent antioxidant (Clark et al., 2000). Nrf2 promotes the transcription of HO-1, enhancing cellular antioxidant capacity (Loboda et al., 2016). The results indicate that Nrf2 positively regulates SLC7A11, TXNRD1, GSS, GCL, GSR, GPX4, FSP1, PPP enzymes, and HO-1, enhancing antioxidant capacity and inhibiting ferroptosis (Figure 1D).

Besides playing a role in the antioxidant system of ferroptosis, Nrf2 also influences iron and lipid metabolism processes (Figure 1D). FTL and FTH1, the light and heavy chains of FT, are regulated by Nrf2. FTL stabilizes FT, while FTH1 has ferroxidase activity, converting Fe^2+^ to Fe^3+^ and storing it in the FT core, sequestering excess free iron and limiting Fe^2+^'s involvement in lipid redox reactions. Nrf2’s regulation of FTL and FTH1 increases Fe^2+^ storage in FT, lowering the LIP (Kerins and Ooi, 2017). HERC2, an E3 ubiquitin ligase, degrades NCOA4, inhibiting FT autophagy and reducing free iron levels. Nrf2 upregulates HERC2 to combat ferroptosis (Anandhan et al., 2023). Additionally, Nrf2 upregulates FPN1, promoting iron export, reducing intracellular iron concentration, and preventing iron overload and oxidative stress (Zhang L. et al., 2021).

## 3 Ferroptosis as a driver of central nervous system diseases and its therapeutic implications

As research on ferroptosis advances, its therapeutic potential has garnered widespread attention. The CNS is particularly vulnerable to lipid metabolism abnormalities and oxidative stress due to its high lipid content and relatively low levels of antioxidant enzymes (Salim, 2017; Lee KH. et al., 2020). Disruptions in iron metabolism, lipid metabolism, and the collapse of cellular oxidative defense systems can accelerate the production of lipid peroxides, damaging the CNS and leading to secondary injuries in acute CNS injuries and NDDs (Ratan, 2020). Consequently, ferroptosis inhibitors have shown significant therapeutic potential in treating these conditions.

### 3.1 Mechanisms of ferroptosis in ischemic stroke

**FIGURE 2:**
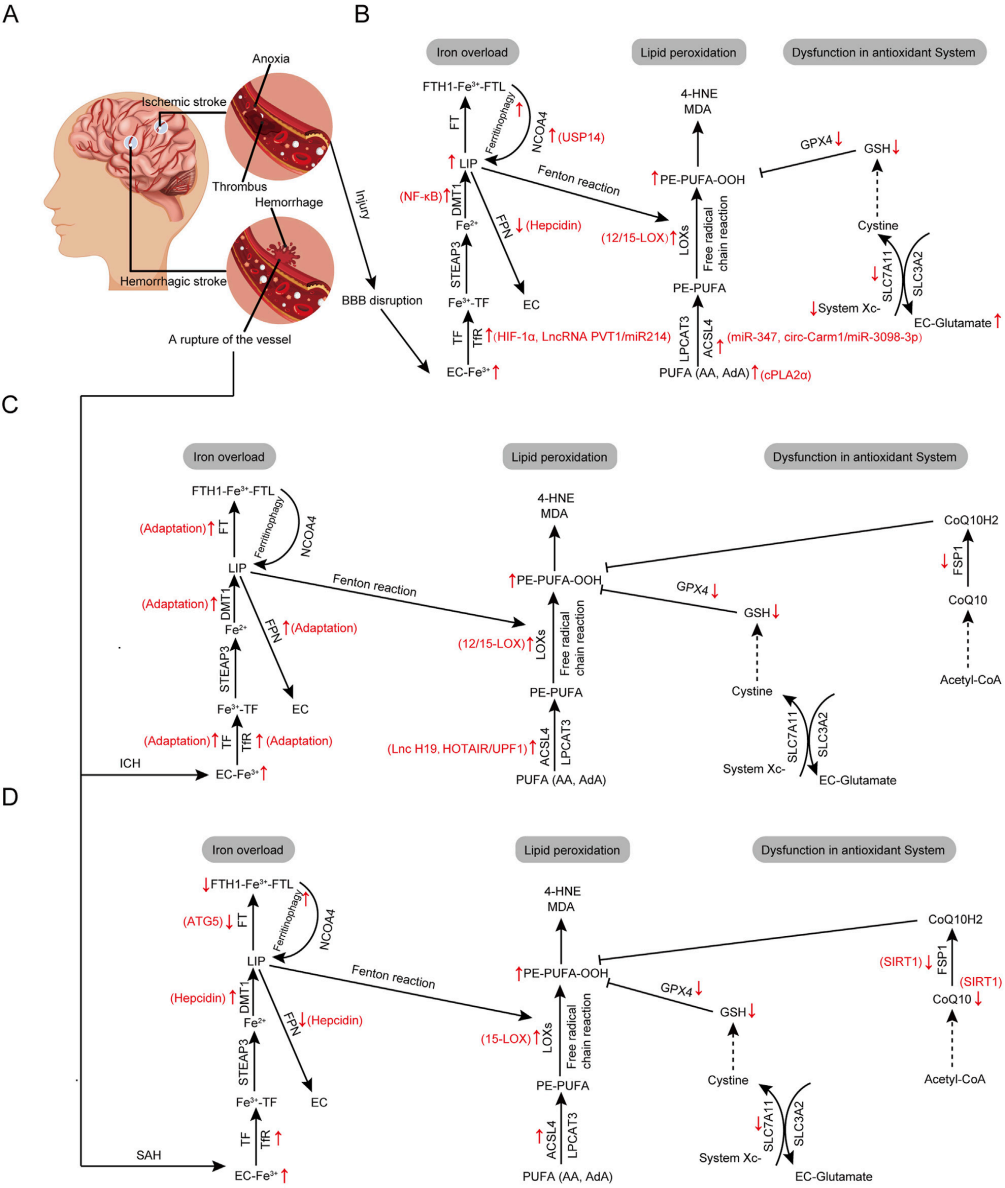
Core regulatory molecules and signaling pathways of ferroptosis in stoke. **(A)** The pathogenesis of ischemic and hemorrhagic stroke. **(B)** Alterations in ferroptosis pathways in ischemic stroke. **(C)** Alterations in ferroptosis pathways in intracerebral hemorrhag. **(D)** Alterations in ferroptosis pathways in subarachnoid hemorrhage. Third-party elements were sourced under CC BY (modifiable, commercial use with attribution). CC BY materials originate from the Freepik library (https://www.freepik.com/).

Stroke, defined as an acute focal CNS injury caused by vascular events, results in neurological deficits. It is classified into IS and HS, with IS being the predominant type, accounting for about four-fifths of all strokes (Figure 2A; Benjamin et al., 2019). The sudden onset of focal or diffuse neurological impairment is a primary cause of stroke-related death and disability. Modulating and intervening in neuronal cell death post-stroke are crucial for reducing neurological damage and improving long-term outcomes (Zhao et al., 2022). IS is caused by the occlusion or narrowing of cerebral blood vessels, resulting in neuronal injury and necrosis in the ischemic core and surrounding penumbra due to insufficient nutrient supply and metabolic disturbances (Figure 2A; Yang and Liu, 2021). The abrupt depletion of glucose and oxygen in local brain tissue is a primary cause of neuronal damage in IS (Babu et al., 2022). Intravenous thrombolytic therapy can rapidly salvage the ischemic penumbra and restore cerebral blood flow (Sylaja and Demchuk, 2008; Saver et al., 2015), but subsequent cerebral ischemia-reperfusion injury (CIRI) exacerbates the lesion area with increased ROS and inflammatory responses (Zhang Q. et al., 2022). Additionally, symptomatic blood flow recovery and reoxygenation can also cause damage, making the prevention of secondary cell deaths crucial (Eltzschig and Eckle, 2011). The mechanisms of neuronal injury and death post-ischemia are complex, with ferroptosis playing a crucial role (Hu et al., 2022; Cui et al., 2021; Zheng et al., 2022). Research indicates that, compared to the ischemic phase, significant changes in ferroptosis markers are more prominently observed during reperfusion, with prolonged reperfusion increasing ACSL4, iron, and MDA levels, and decreasing GPX4 levels (Tang LJ. et al., 2021).

#### 3.1.1 Mechanism of iron overload-mediated ferroptosis involved in ischemic stroke

Iron overload is considered a major cause of ferroptosis following IS (Figure 2B; Chi et al., 2000; Selim and Ratan, 2004). Ischemia leads to endothelial cell damage, blood-brain barrier (BBB) disruption, and increased permeability, allowing a large influx of iron into the brain parenchyma (Bu et al., 2021). This results in local iron metabolism disorders, creating an intracellular environment of iron overload that promotes the generation of L-OOHs, which in turn cause nucleic acid, protein, and membrane damage, ultimately triggering ferroptosis (Tuo et al., 2017). In middle cerebral artery occlusion (MCAO) models simulating IS, significant iron deposition has been observed in the ischemic brain tissue; supplementing iron in MCAO rats exacerbates infarct severity, while iron chelators reduce infarct size (Tuo et al., 2017; Abdul et al., 2021).

Research indicates that nearly all proteins involved in iron metabolism undergo changes during ischemic stroke, leading to iron overload (Lan et al., 2020; Cojocaru et al., 2013; Tang et al., 2020). These iron metabolic changes are associated with poor prognosis in IS patients (Gill et al., 2018; Dávalos et al., 1994; Dávalos et al., 2000). As ischemic conditions increase hypoxia-inducible factor-1α (HIF-1α) levels, which promote TfR1 expression, the upregulation of TfR1 enhances cellular iron uptake (Hirayama and Koizumi, 2017; Yang et al., 2018; Ding et al., 2011). Additionally, long non-coding RNA (LncRNA) PVT1 is elevated in the plasma of CIRI patients and can regulate TfR1 expression through the LncRNA PVT1/miR214 axis, inducing ferroptosis (Lu et al., 2020). Ingrassia et al. observed increased expression of DMT1, regulated by nuclear factor kappa-light-chain-enhancer of activated B cells (NF-κB), in both animal and cellular models of IS, which is associated with increased neuronal cytoplasmic iron influx (Ingrassia et al., 2012). The increased intracellular iron ions further bind with FT. However, NCOA4, upregulated in IS via deubiquitination by USP14, may mediate the autophagy of FT, thereby increasing cytosolic LIP levels and further driving ferroptosis (Abdul et al., 2021). Meanwhile, iron efflux is inhibited after IS. Ding et al. showed that hepcidin is significantly upregulated in IS patients, promoting FPN1 degradation and blocking iron efflux (Ding et al., 2011; Słomka et al., 2015).

#### 3.1.2 Mechanism of lipid peroxidation-mediated ferroptosis involved in ischemic stroke

As outlined, lipid peroxidation, particularly the conversion of PUFAs in the phospholipid bilayer to L-OOHs, is a crucial mechanism of ferroptosis and plays a significant role in the development of IS. The brain is rich in PUFAs, particularly AA, making it susceptible to lipid peroxidation during ischemic stroke (Kloska et al., 2020). Cytosolic phospholipase A2α (cPLA2α) is a Ca2+-dependent enzyme that plays a crucial role in initiating AA metabolism (Muralikrishna Adibhatla and Hatcher, 2006). Elevated expression of cPLA2α has been observed in IS patients, correlating with increased severity of injury and infarct size (Cui et al., 2021). Studies have indicated that cPLA2α is overactivated in ischemic brain tissue via N-methyl-D-aspartate (NMDA) receptor/Ca2+ and thrombin pathways, promoting the mobilization of AA (Xu et al., 2024; Murakami and Kudo, 2002; Tuo et al., 2022).

ACSL4, an essential enzyme for the pre-oxidation preparation of PUFAs, plays a critical role in determining sensitivity to ferroptosis (Cui et al., 2021; Doll et al., 2017). Gubern et al. found that miR-347 is upregulated in the permanent middle cerebral artery occlusion (pMCAO) model, with Acsl4 upregulation following miR-347 overexpression potentially inducing neuronal death. This suggests that the miR-347/ACSL4 axis may promote lipid peroxidation and mediate ferroptosis (Gubern et al., 2013). Additionally, ACSL4 expression is regulated by circular RNAs (circRNAs). Circ-Carm1, highly expressed in oxygen-glucose deprivation/reperfusion (OGD/R)-induced cells, may promote lipid peroxidation and mediate ferroptosis in acute cerebral infarction through the circ-Carm1/miR-3098-3p/ACSL4 axis (Mao and Liu, 2022). Tuo et al. found that knocking out ACSL4 does not affect cortical blood flow after middle cerebral artery occlusion/reperfusion (MCAO/R) in rats, but it reduces infarct volume and mitigates neural damage by inhibiting ferroptosis (Tuo et al., 2022). Chen et al. demonstrated that ACSL4 inhibition alleviates ferroptosis-related lipid peroxidation, improves neurological function, and reduces infarct volume after stroke (Chen J. et al., 2021). Moreover, LOX, particularly the 12/15-LOX subtype, are key enzymes that catalyze the formation of L-OOHs from PEs, directly oxidizing PUFA-containing lipid membranes through enzymatic pathways (Singh and Rao, 2019; Li et al., 2018). Research by Jin et al. revealed that excessive 12/15-LOX expression post-stroke leads to neuronal death and blood-brain barrier compromise, with 12/15-LOX inhibitors enhancing neurological function (Singh and Rao, 2019; Jin et al., 2008; van Leyen et al., 2006; Zhang et al., 2004). These findings highlight lipid peroxidation-related enzymes, particularly 12/15-LOX and ACSL4, as novel therapeutic targets for treating secondary brain injury post-stroke (Figure 2B).

#### 3.1.3 Mechanism of antioxidant system dysfunction-mediated ferroptosis involved in ischemic stroke

In addition to excessive lipid peroxidation and iron accumulation, ferroptosis-inhibiting pathways are also suppressed in IS (Figure 2B; Hu et al., 2024). The System Xc-/GSH/GPX4 axis is pivotal in clearing lipid peroxides during ferroptosis. System Xc-, a glutamate/cystine antiporter, regulates intracellular cystine and glutamate exchange, crucial for synthesizing GSH and GPX4. Numerous studies indicate that the expression of SLC7A11, GSH, and GPX4 decreases following CIRI (Guan et al., 2021; Shi et al., 2022; Zhu et al., 2024). During IS, elevated extracellular glutamate levels (Speer et al., 2013; Jabaudon et al., 2000), due to decreased uptake, increased vesicular release, and non-vesicular release, inhibit System Xc-, hindering cystine-glutamate exchange and thereby suppressing GSH production and GPX4 function, which triggers ferroptosis (Fan et al., 2023; Zhang et al., 2021d). The expression of SLC7A11, a crucial component of System Xc-, decreases in neurons following OGD/R. This reduction impairs lipid peroxide clearance and increases L-OOHs accumulation, leading to neuronal death (Yuan et al., 2021). Additionally, GSH acts as an endogenous ferroptosis inhibitor. In oxidative stress disorders, including stroke, GSH levels are depleted, and reduced brain GSH levels are associated with increased stroke risk (Zhang et al., 2021d). Conversely, exogenous GSH supplementation can alleviate IS by increasing striatal dopamine levels, which upregulate GSH synthase and homocysteine, thereby enhancing GSH’s therapeutic efficacy in the brain (Wang H. et al., 2022; Liu Y. et al., 2020). Research indicates that N-acetylcysteine (NAC) inhibits ferroptosis induced by GSH depletion by acting as a cysteine precursor to increase GSH levels and has been clinically approved for treating acute IS (Kalyanaraman, 2022; Komakula et al., 2024; Sabetghadam et al., 2020). GPX4, integral in inhibiting lipid peroxidation and closely linked with ferroptosis in stroke patients, shows significantly reduced protein expression in both *in vivo* and *in vitro* IS models (Zhu et al., 2022). GPX4 utilizes GSH to reduce L-OOHs to their corresponding alcohols (L-OHs), protecting cells from oxidative damage; thus, boosting GSH synthesis mitigates neurological damage in IS (Zhang et al., 2021d; Liu Y. et al., 2020). Li et al. demonstrated that baicalin, a major component of Scutellaria, prevents ferroptosis damage in transient middle cerebral artery occlusion (tMCAO) mice or OGD/R cells by enhancing GPX4 expression (Li M. et al., 2022). Liu et al. found that the free radical scavenger edaravone reduces infarct volume and dysfunction by increasing GSH levels and GPX4 expression, thereby exerting anti-ferroptosis effects (Liu W. et al., 2022).

#### 3.1.4 Ferroptosis in cerebral ischemia-reperfusion injury after ischemic stroke

During cerebral ischemia-reperfusion, excessive ROS are generated, closely linking CIRI with ferroptosis activation by ROS (Liu et al., 2024b; Tian et al., 2024). The mechanisms of excessive ROS production during the CIRI process include: (1) Mitochondrial dysfunction during ischemia reduces electron transport chain efficiency, increasing free radical generation; reperfusion further elevates ROS production (Zhang Q. et al., 2022; Zweier and Talukder, 2006). (2) ATP depletion under hypoxia generates xanthine, converting xanthine dehydrogenase to xanthine oxidase; reperfusion then leads to massive ROS generation by xanthine oxidase (Li et al., 2011). (3) Inflammation and chemokine production during ischemia recruit and activate neutrophils, which significantly increase oxygen consumption and ROS production during reperfusion, a process known as “respiratory burst” (Mittal et al., 2014; Francisco and Del Re, 2023). (4) Membrane dysfunction during ischemia causes Ca^2+^ overload, activating phospholipase A_2_, which degrades phospholipids into AA, generating ROS through COX pathways (Tang LJ. et al., 2021; Hassannia et al., 2019; Zhang et al., 2020a). (5) Sympathoadrenal activation during reperfusion releases catecholamines and induces acidosis, increasing ROS (Guo et al., 2023). The brain, deficient in catalase, has reduced antioxidant defenses during ischemia, and reperfusion exacerbates ROS production, leading to oxidative imbalance (Lee KH. et al., 2020; Singh et al., 2019). The high PUFA content and non-regenerative nature of neurons, make the brain particularly susceptible to ferroptosis during CIRI, resulting in neuronal damage (Sublette et al., 2024). Additionally, during CIRI, the BBB is damaged, leading to iron homeostasis imbalance in the brain, which further drives the occurrence of ferroptosis (Liu et al., 2024c; Chen X. et al., 2022).

### 3.2 Mechanisms of ferroptosis in hemorrhagic stroke

Although HS accounts for only about 20% of all strokes, its mortality and disability rates are higher than those of IS (Benjamin et al., 2019; Lim et al., 2020). HS is an acute condition caused by the sudden rupture of specific brain vessels, leading to bleeding within the brain parenchyma (Figure 2A), known as ICH, or into the subarachnoid space, known as SAH. ICH accounts for 80% of HS cases, while SAH accounts for the remaining 20% (Donkor, 2018). These conditions involve two phases: primary brain injury due to mechanical damage from the hematoma, increased intracranial pressure, and secondary cerebral infarction; and secondary pathophysiological events from blood components and metabolites, including BBB disruption, neuroexcitatory events, ionic imbalances, oxidative stress, neuroinflammation, and cell death (Magid-Bernstein et al., 2022). Recent studies confirm the presence of ferroptosis in HS and highlight key targets regulating this process (Figures 2C, D; Alim et al., 2019; Cao et al., 2021). Crucially, inhibiting or downregulating ferroptosis in neurons shows promise as a potential therapy for HS (Chang et al., 2014; Qu et al., 2021; Ren et al., 2022; Shao et al., 2019).

#### 3.2.1 Mechanism of iron overload-mediated ferroptosis involved in intracerebral hemorrhage

Primary injury in ICH occurs within hours after ICH, where ruptured blood vessels form localized hematomas that directly damage brain tissue, disrupt neuronal and fiber connections, and cause neurological deficits. The mass effect of the hematoma significantly increases intracranial pressure, compressing surrounding brain tissue and neural tracts, potentially leading to brain herniation (Wilkinson et al., 2018). Secondary injury also begins within the first few hours of ICH and peaks around 3 days, involving blood-brain barrier disruption, cerebral edema, inflammation, Hb degradation products toxicity, and cell death (Kearns et al., 2021; Loan et al., 2022). After ICH, Hb/heme/iron is recognized as a major contributor to delayed cerebral edema and irreversible neuronal damage, playing a crucial role in lipid ROS production (Xiong et al., 2014). Studies have found that ferroptosis, occurs after ICH and contributes to neuronal death. Therefore, inhibiting ferroptosis may protect neurons from secondary injury (Li Q. et al., 2017; Wan et al., 2019; Chaudhary et al., 2013).

The accumulation of blood components in the damaged area is a key pathological feature of hemorrhagic conditions, with hemoglobin release from lysed red blood cells and subsequent degradation into heme and free iron being major contributors to iron overload in ICH (Wang and Doré, 2007). After ICH, activated microglia and macrophages in the damaged area engulf hemoglobin from lysed red blood cells, degrade it, and release iron (Wan et al., 2019; DeRosa and Leftin, 2021). Excess extracellular free iron enters neurons through the TF-TfR pathway, causing iron overload and inducing subsequent lipid peroxidation (Wan et al., 2019; Andrews, 2000). Cerebrospinal fluid TF saturation is much higher than plasma, potentially compromising iron regulation and predisposing brain cells to ferroptosis under iron overload conditions (Pagani et al., 2015; Baringer et al., 2022). Additionally, ICH increases levels of iron-binding proteins and TF, leading to substantial Fe^3+^ endocytosis into brain cells (Chaudhary et al., 2013; DeGregorio-Rocasolano et al., 2019). Post-hemorrhage, brain cell metabolism is disrupted, blood pH drops, inducing Fe^3+^ dissociation from complexes, and Fe^3+^ is reduced to Fe^2+^ by iron reductases (Duck and Connor, 2016). The Fe^2+^ is either utilized by cells, stored in FT, or exported via Fpn1 to maintain systemic iron balance. After a brain hemorrhage, the accumulation of Fe^2+^ creates an unstable LIP, which can participate in Fenton reactions and induce ferroptosis (Sun et al., 2022).

In 2004, Nakamura et al. discovered iron deposition in the basal ganglia of a rat ICH model (Nakamura et al., 2004). Furthermore, following ICH, the levels of brain iron-handling proteins, including DMT1, FPN, ferritin, TF, and TfR, significantly increase, indicating the occurrence of iron overload and the neuronal response (Wu et al., 2003). Moreover, studies have demonstrated that iron chelators effectively remove excess iron. After crossing the BBB, iron chelators form stable complexes with ferric iron, reducing free radical production (Yeatts et al., 2013). *In vivo* ICH models show that iron chelators can reduce cerebral edema, neurological deficits, and brain atrophy (Nakamura et al., 2004; Okauchi et al., 2010). Targeting iron overload is crucial for treating ICH, and targeting FPN has shown potential in reducing neuronal death by inhibiting ferroptosis in aged ICH (Bao et al., 2020). Thus, iron overload-induced ferroptosis significantly contributes to secondary injury in ICH, exacerbating oxidative stress and lipid peroxidation (Figure 2C). This damage can be mitigated by using iron chelators and targeting iron metabolism-related proteins, which helps reduce neuronal death.

#### 3.2.2 Mechanism of lipid peroxidation-mediated ferroptosis involved in intracerebral hemorrhage

During ICH, excess iron catalyzes oxidative stress and lipid peroxidation of cell membranes, ultimately leading to cell death. lipid peroxidation damages proteins, DNA, and lipid membranes, thereby activating ferroptosis (Figure 2C; Fang et al., 2022; Xu et al., 2023). Reducing ferroptosis by inhibiting lipid peroxidation has become a crucial and effective target for protecting against ICH(202). Edaravone, as a free radical scavenger, reduced brain edema and inhibited lipid peroxidation following intracerebral hemorrhage in rats (Chen Z. et al., 2014). ACSL4 is a key lipid-metabolizing enzyme that induces lipid peroxidation and ferroptosis (Cheng et al., 2020). Recent studies show that ACSL4 is highly expressed in brain tissue around hematomas in ICH mice and plays a key role in ferroptosis (Chen B. et al., 2021; Jin et al., 2021; Pan et al., 2022). Additionally, ACSL4 is regulated by LncRNAs, with LncRNA H19 upregulating ACSL4 expression during ICH (Chen B. et al., 2021). Jin et al. demonstrated that HOTAIR binds to UPF1, which promotes the degradation of ACSL4, thereby reducing ferroptosis. Therefore, targeting the HOTAIR/UPF1/ACSL4 axis is an effective strategy to inhibit ferroptosis and reduce neuronal death in ICH (Jin et al., 2021). LOX plays a critical role in the enzymatic pathway of lipid peroxidation involved in ferroptosis during ICH (Bai et al., 2020). 12/15-LOX inhibitors, which inhibit lipid peroxidation, reduced hemorrhagic transformation in warfarin-treated mice after experimental stroke and contribute to the treatment of ICH (Bai et al., 2020; Liu et al., 2017). Previous studies have shown that NAC can neutralize toxic lipids produced by AA-dependent 5-LOX activity, preventing heme-induced ferroptosis and ultimately improving outcomes in mice after ICH (Karuppagounder et al., 2018). Additionally, baicalein, a non-specific inhibitor of 12/15-LOX, significantly increased ferroptosis-related markers after ICH (Duan et al., 2021). Thus, inhibiting lipid peroxidation to deactivate ferroptosis has emerged as a significant potential therapeutic target for ICH (Zhou SY. et al., 2020).

#### 3.2.3 Mechanism of antioxidant system dysfunction-mediated ferroptosis involved in intracerebral hemorrhage

Various antioxidant pathways can inhibit ferroptosis and may serve as effective targets for protecting against ICH (Figure 2C). The System Xc-/GSH/GPX4 axis is one of the most extensively studied antioxidant pathways. Following ICH, iron accumulation and excessive lipid peroxides trigger ferroptosis. After ICH, ferroptosis is caused by GSH synthesis defects and reduced GPX4 levels (Zhang Z. et al., 2018; Wang S. et al., 2018). Studies show that GPX4 levels in neurons significantly decrease after ICH. Inhibiting GPX4 worsens brain injury, while upregulating GPX4 protects neurons from ferroptosis and improves neurological function in rats with ICH (Zhang Z. et al., 2018). Delivering selenium to the brain promotes the expression of the antioxidant GPX4, inhibits neuronal ferroptosis, and improves function in HS models (Alim et al., 2019). Systemic administration of NAC, an approved cysteine prodrug, increases cellular cysteine and GSH synthesis, inhibiting neuronal ferroptosis after ICH (Zille et al., 2017). Post-ICH, significantly decreased GSH levels can be restored with GSH treatment, reducing brain edema and alleviating neurological damage in ICH mice (Diao et al., 2020). In summary, most studies indicate that GSH levels and GPX4 expression are downregulated in ICH, but ferroptosis can be reversed with antioxidant drugs like dauricine or microRNA, providing neuroprotection (Peng et al., 2022).

A recent study shows that FSP1 levels are significantly reduced in the brain tissue surrounding hematomas in ICH mice, a change reversible with dexpramipexole treatment (Wang B. et al., 2022). However, the detailed variation patterns and potential mechanisms of the FSP1/CoQ10/NADPH axis still require further investigation.

#### 3.2.4 Mechanisms of ferroptosis in subarachnoid hemorrhage

When intracranial blood vessels rupture, blood enters the subarachnoid space, causing SAH (Ducros and Bousser, 2013; van Gijn et al., 2007). About 85% of non-traumatic SAH cases are due to ruptured aneurysms, while the remaining 15%–20% result from various other causes with often indeterminate bleeding mechanisms (Steiner et al., 2013). Regardless of the cause, SAH leads to high mortality and disability rates (Fang et al., 2020; Nieuwkamp et al., 2009). Additionally, SAH patients face a high risk of complications, including early brain injury (EBI) and delayed brain injury (DBI) (Chen J. et al., 2014). Within 72 h of SAH onset, the body undergoes pathological changes such as BBB disruption, cerebral edema, and neuronal damage defined as EBI, which is closely associated with poor prognosis (Nieuwkamp et al., 2009; Zhang et al., 2020b). EBI can lead to vasospasm-related delayed cerebral ischemia, occurring 3–4 days after the initial hemorrhage, which further worsens neurological function and causes DBI (van Lieshout et al., 2018; Petridis et al., 2017; Weiland et al., 2019; Dabbagh Ohadi et al., 2024). Recent studies have confirmed that ferroptosis is associated with SAH (Figure 2D), and subsequent research has demonstrated its occurrence in animal and *in vitro* models of SAH (Cao et al., 2021; Qu et al., 2021; Li S. et al., 2021; Gao et al., 2022).

Increased iron levels and FT degradation are major causes of brain injury after SAH. Deferoxamine, an iron chelator, reduces brain injury, indicating iron overload as a crucial trigger for ferroptosis and providing neuroprotective insights (Xi et al., 2006). During SAH, red blood cells enter the subarachnoid space, rapidly increasing extracellular iron ions (van Gijn et al., 2007). These ions bind with TF and TfR to form a complex, entering brain cells. Through STEAP3-mediated reduction and DMT1-mediated transport, Fe^2+^ is released into the cytoplasm. Some Fe^2+^ is oxidized to Fe^3+^ and stored as FT-bound inert iron, while the remaining Fe^2+^ forms a LIP, inducing lipid peroxidation via the Fenton reaction, or is exported via FPN (Liu Q. et al., 2021; Masaldan et al., 2019; Mancardi et al., 2021). In SAH rat models, TfR levels significantly upregulate at 24 h post-SAH (Li et al., 2021c). Yuan et al. noted TfR and DMT1 levels increased within 6 h in EBI (Yuan et al., 2022). Zhang et al. reported hepcidin and DMT1 upregulation in EBI post-SAH; DMT1 inhibitor ebselen reduced intracellular iron and ferroptosis (Zhang H. et al., 2021). Ferritinophagy is involved in EBI post-SAH. Liang et al. reported SAH-induced ferritinophagy reduced FTH1, increasing LIP and leading to ferroptosis (Liang Y. et al., 2022). Inhibiting autophagy-related gene 5 (ATG5), which regulates ferritinophagy, increased FT, decreased LIP and lipid peroxidation, alleviating SAH-induced ferroptosis and improving outcomes (Liang Y. et al., 2022; Zhao et al., 2014). FPN is a key protein reducing intracellular iron (Trujillo-Alonso et al., 2019). In EBI, the upregulation of hepcidin leads to the degradation of FPN, resulting in increased intracellular iron (Zhang H. et al., 2021; Nemeth et al., 2004). Additionally, Li et al. found that Fer-1 treatment upregulated FPN, reduced iron levels, mitigated lipid peroxidation, inhibited ferroptosis, and improved neurological function post-SAH (Li et al., 2021c). These findings indicate that iron overload and iron metabolism proteins mediate ferroptosis, presenting a potential breakthrough for treating EBI post-SAH. Additionally, studies show that iron chelators play a role in vasospasm-induced secondary ischemia, and their mechanism might involve inhibiting iron-induced ROS and lipid peroxidation, indicating that iron overload may influence the development of DBI. (Utkan et al., 1996). Subsequent studies repeatedly confirmed the effectiveness of iron chelators in reducing vasospasm (Utkan et al., 1996; Luo et al., 1995).

Lipid peroxidation following SAH significantly damages biological membranes and lipoproteins, mediating ferroptosis and leading to secondary neuronal death (Chen J. et al., 2022). Cao et al. observed ferroptosis in SAH via electron microscopy, noting mitochondrial shrinkage, compressed membrane density, reduced cristae, and ruptured outer membranes (Cao et al., 2021). Li et al.'s quantitative analysis showed reduced mitochondrial area in the SAH group, while the SAH + Fer-1 group exhibited improved mitochondrial morphology (Li et al., 2021c). ACSL4 and LOX play key roles in lipid peroxidation; ACSL4 incorporates PUFAs into phospholipids, and LOX catalyzes their oxidation, leading to lipid peroxidation (Liang D. et al., 2022). Qu et al. found that in a SAH rat model, ACSL4 expression significantly increased in EBI; inhibiting ACSL4 with siRNA reduced inflammation, BBB damage, oxidative stress, brain edema, behavioral and cognitive deficits, and increased neuron survival (Qu et al., 2021). ACSL4 exacerbates brain injury via lipid metabolism and is a key predictor of ferroptosis in SAH (Yuan et al., 2022; Huang et al., 2022). During SAH, 15-LOX is highly expressed in microglia, and reducing its levels with drugs can inhibit ferroptosis (Gao et al., 2022). The 15-LOX inhibitor baicalein reduces ferroptosis and alleviates EBI post-SAH (Zhang HB. et al., 2020). Research on ACSL4 and LOXs may elucidate the mechanisms of secondary injury post-SAH and offer new therapeutic strategies.

Inhibition of peroxide clearance promotes ferroptosis, with the System Xc-/GSH/GPX4 axis being a key antioxidant pathway in this process. Before ferroptosis was conceptualized, studies had already detected reduced GPX activity and GSH levels in the cerebrospinal fluid (CSF) of patients (Suzuki et al., 1983). Similarly, decreased GPX levels were observed in the hippocampus of SAH rat models (Sakaki et al., 1986). These findings suggest an antioxidant system imbalance in SAH. Subsequent drug supplementation experiments indicated that enhancing GSH/GPX antioxidant activity could treat SAH and provide neuroprotection (Handa et al., 2000; Ayer et al., 2008; Lu et al., 2009). After the concept of ferroptosis was established, research focused on GPX4, a key enzyme in its antioxidant system. Gao et al. reported that GPX4 levels significantly decrease in a rat model of EBI after SAH (Gao et al., 2020). Adenoviral overexpression of GPX4 suppressed lipid peroxidation and ferroptosis *in vivo* and *in vitro*, improving brain edema and neurological dysfunction within 24 h post-SAH (Gao et al., 2020; Chen et al., 2024). Li et al. found that GSH levels and GPX4 activity significantly decreased in the cerebral cortex of rats post-SAH. Fer-1, a ferroptosis inhibitor, effectively increased GSH and GPX4 levels, indicating that Fer-1 prevents ferroptosis in EBI by inhibiting neuronal lipid peroxidation (Li et al., 2021c). As a key regulator of GSH synthesis, SLC7A11 was impaired in SAH models, but its protein reduction was less pronounced than that of GPX4 (Liu Z. et al., 2022). Guan et al. reported that FSP1 and CoQ10 levels significantly decrease in in vivo and *in vitro* SAH models, suggesting that FSP1/CoQ10-mediated ferroptosis may contribute to EBI after SAH. They emphasized that activation of the epigenetic regulator Sirtuin 1 (SIRT1) reduces neuronal ferroptosis in SAH by upregulating FSP1 and CoQ10B expression (Yuan et al., 2022).

### 3.3 Mechanisms of ferroptosis in spinal cord injury

**FIGURE 3:**
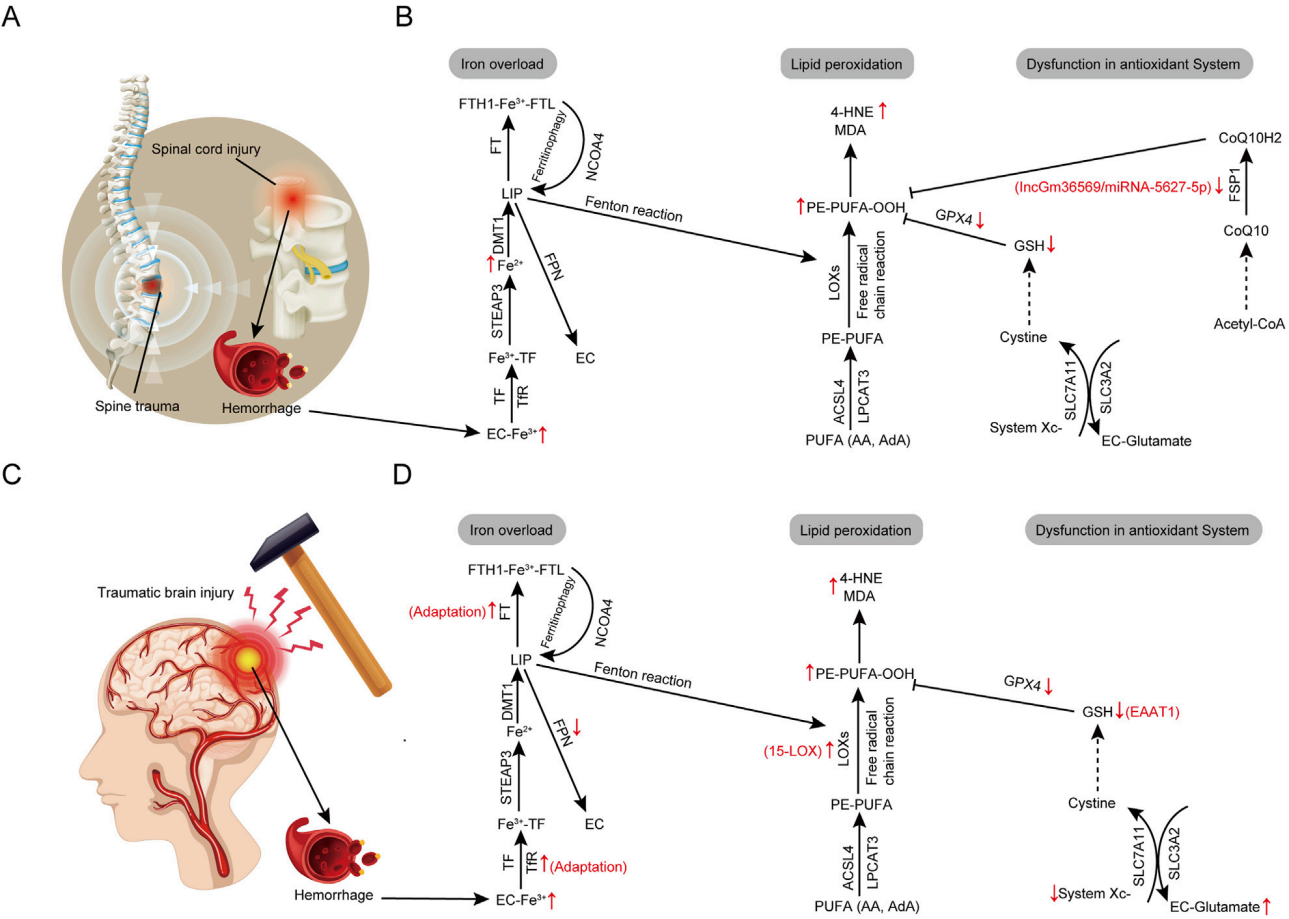
Core regulatory molecules and signaling pathways of ferroptosis in acute central nervous system trauma. **(A)** The pathogenesis of spinal cord injury. **(B)** Alterations in ferroptosis pathways in spinal cord injury. **(C)** The pathogenesis of traumatic brain injury. **(D)** Alterations in ferroptosis pathways in traumatic brain injury. Third-party elements were sourced under CC BY (modifiable, commercial use with attribution). CC BY materials originate from the Freepik library (https://www.freepik.com/).

SCI is the most severe complication of spinal trauma, often leading to the loss of sensory, motor, and autonomic functions (Figure 3A). The pathological process of SCI consists of two phases: primary and secondary injuries. Primary injuries occur instantaneously and are proportional in severity to the trauma, characterized by localized impact and brief duration, typically irreversible by external means (Sofroniew, 2018). Secondary injuries are induced by a variety of physical and chemical factors such as local bleeding, edema, oxidative stress, and inflammation (Ahuja et al., 2017). These injuries have a wider impact and longer duration, significantly affecting the survival of residual neuronal and neurovascular units, and hindering neuron regeneration and axon restoration. Consequently, mitigating secondary injuries post-SCI is a critical focus of ongoing research. Over time, interactions among various cells within the spinal cord tissue, including astrocytes, neurons, microglia, and oligodendrocytes, along with a series of biochemical and physiological changes, initiate secondary injury events leading to ferroptosis (Figure 3B). This process generates an abundance of ROS, ion dysregulation (including but not limited to iron ions), glutamate-mediated excitotoxicity, and immune-related neurotoxicity. Consequently, effectively blocking and reversing these secondary injuries is crucial for inhibiting ferroptosis (Ambrozaitis et al., 2006; Visavadiya et al., 2015).

During the early stages of SCI, substantial hemorrhage, red blood cell aggregation, cellular rupture, and hemolysis lead to a significant release of iron ions (Figure 3B; Yin et al., 2024; Wang Z. et al., 2022). These iron ions are taken up by cells through TF and its receptors, leading to intracellular iron accumulation, which catalyzes iron-dependent Fenton reactions, producing excessive ROS that cause lipid peroxidation and membrane damage (Meng et al., 2017; Gong et al., 2022; Feng et al., 2021). Meng et al. observed significant changes in ferroptosis markers within the spinal cord tissues of SCI rats, and transmission electron microscopy revealed characteristic mitochondrial alterations associated with ferroptosis, confirming its role in SCI (Meng et al., 2017). Additionally, iron binds to GSH, reducing the available reduced GSH and inactivating GPX4. This enzyme’s inactivation, combined with the depletion of reduced GSH, escalates lipid peroxidation of the cell membrane, ultimately triggering ferroptosis (Yang et al., 2014). Studies have also demonstrated that iron overload and lipid peroxidation are key inducers of ferroptosis in the pathophysiology of SCI (Figure 3B). *In vitro* experiments adding ferrous ions to spinal neuronal cultures showed that the quantity of lipid peroxidation metabolites correlates directly with iron levels and is positively associated with neuronal inactivation (Galluzzi et al., 2012). Furthermore, administering ferroptosis inhibitors such as deferoxamine (DFO) protects neurons and enhances recovery of motor functions, suggesting that inhibiting ferroptosis can facilitate recovery after SCI (Yao et al., 2019). Lipid peroxidation plays a pivotal role in secondary spinal cord injury, as evidenced by a marked increase in lipid peroxidation markers such as 4-HNE in the injured spinal tissue (Springer et al., 1997). Additionally, the spinal cord contains high levels of PUFAs, which are prone to oxidation following SCI, providing a basis for iron-dependent lipid peroxidation and promoting ferroptosis (Baazm et al., 2021). In SCI progression, the inhibition of antioxidant pathways is crucial for promoting ferroptosis. GPX4 downregulation was observed in the acute phase of an SCI animal model, and another study found that GPX4 knockout-induced degeneration of spinal motor neurons exhibits ferroptosis, with vitamin E supplementation delaying paralysis and death in GPX4 knockout mice (Chen et al., 2015; Zhou H. et al., 2020). Additionally, a study found that in SCI animal models, the IncGm36569/miRNA-5627-5p/FSP1 axis was inhibited through molecular sponge action, thereby targeting this axis to inhibit neuronal ferroptosis (Shao et al., 2022). It is evident that the inhibition of antioxidant systems, linked to GPX4 and FSP1 downregulation, plays a crucial role in SCI progression (Figure 3B).

### 3.4 Mechanisms of ferroptosis in traumatic brain injury

TBI is commonly caused by external trauma (Figure 3C), and its stages of damage are like those of SCI, including primary irreversible mechanical damage and secondary injuries (Maas et al., 2022). Previous studies have shown that secondary brain injuries can further lead to neurological deficits and NDDs (Ramos-Cejudo et al., 2018). Therefore, mitigating secondary injuries is a critical strategy in the current treatment of TBI, with the reduction of neuronal death being key to treating secondary injuries (Li LM. et al., 2021). Modulating neuronal ferroptosis to intervene in the secondary injuries of TBI is increasingly becoming a focus of interest in the neuroscience community (Yan et al., 2021).

Post-TBI, localized hemorrhage or microhemorrhages are common, leading to the accumulation of iron ions in brain tissues (Figure 3D). These ions originate from lysed red blood cells within the injury site, released upon hemoglobin breakdown, and subsequently deposit around the brain parenchyma (Huang et al., 2021). Iron accumulation begins early post-TBI and increases over time (Xie et al., 2019). Iron deposition triggers various pathological responses, ultimately exacerbating neuronal tissue damage. As a pro-oxidant, iron-driven lipid peroxidation persists, leading to ferroptosis in affected cells, thus exacerbating secondary brain injury. The process of secondary injury may continue for months to years post-TBI, during which iron deposition areas may expand, intensifying local brain tissue damage (Xie et al., 2019). Higher overlap between iron deposition and lesion areas correlates with more severe damage to neurons and glial cells, resulting in widespread functional impairments (Wehn et al., 2021; Chen et al., 2019). Numerous studies have shown that impaired iron metabolism is linked to TBI. In a controlled cortical injury (CCI) mouse model, iron deposition and abnormal iron metabolism were observed. Intracerebral ventricular injection of the ferroptosis-specific inhibitor Fer-1 significantly reduced iron accumulation and neuronal damage, improving long-term outcomes (Xie et al., 2019). Similarly, TBI induced the expression of TfR and FT while inhibiting the expression of FPN. These findings support the notion of iron accumulation after TBI (Zhang et al., 2019). In a CCI mouse model, an increase in serum PUFAs was found, leading to high levels of lipid peroxidation and making brain tissue more susceptible to ferroptosis (Hogan et al., 2018). Additionally, researchers observed elevated levels of various lipid oxidation markers in the brain tissue or cerebrospinal fluid of TBI patients (Figure 3D; Anthonymuthu et al., 2018). Further evidence from Kenny et al. indicates that the oxidation of PEs, changes in protein expression, and GSH levels are consistent with the activation of ferroptosis following TBI, and that inhibiting 15-LOX significantly reduces ferroptosis in both *in vitro* and *in vivo* studies, suggesting that iron overload-related lipid peroxidation plays an important role in the pathogenesis of TBI(299). Furthermore, the inhibition of antioxidant systems represented by GSH/GPX4 promotes the progression of ferroptosis in TBI. Low or depleted GSH levels are common in TBI. A recent study found that knocking down the excitatory amino acid carrier type 1 (EAAT1) genes to reduce GSH intake significantly increased neuronal cell death in CCI mice (Choi et al., 2016). Additionally, reduced serum GSH in mild TBI patients was linked to posttraumatic epilepsy (Wang HC. et al., 2016). After TBI, increased glutamate release inhibits the normal function of System Xc-, thereby affecting GSH production and making it another ferroptosis pathway to consider in TBI (Guerriero et al., 2015). The observed decrease in GPx4 activity after TBI, along with these findings, suggests that the System Xc−/GSH/GPX4 pathway plays an important role in the activation of ferroptosis following TBI (Figure 3D; Xie et al., 2019; Gaschler et al., 2018). TBI brain damage involves mechanisms of acute cerebrovascular disease and ferroptosis-related chronic NDDs. Therefore, modulating ferroptosis could be a key approach to reducing secondary TBI damage (Xie et al., 2019).

### 3.5 Mechanisms of ferroptosis in Alzheimer’s disease

**FIGURE 4:**
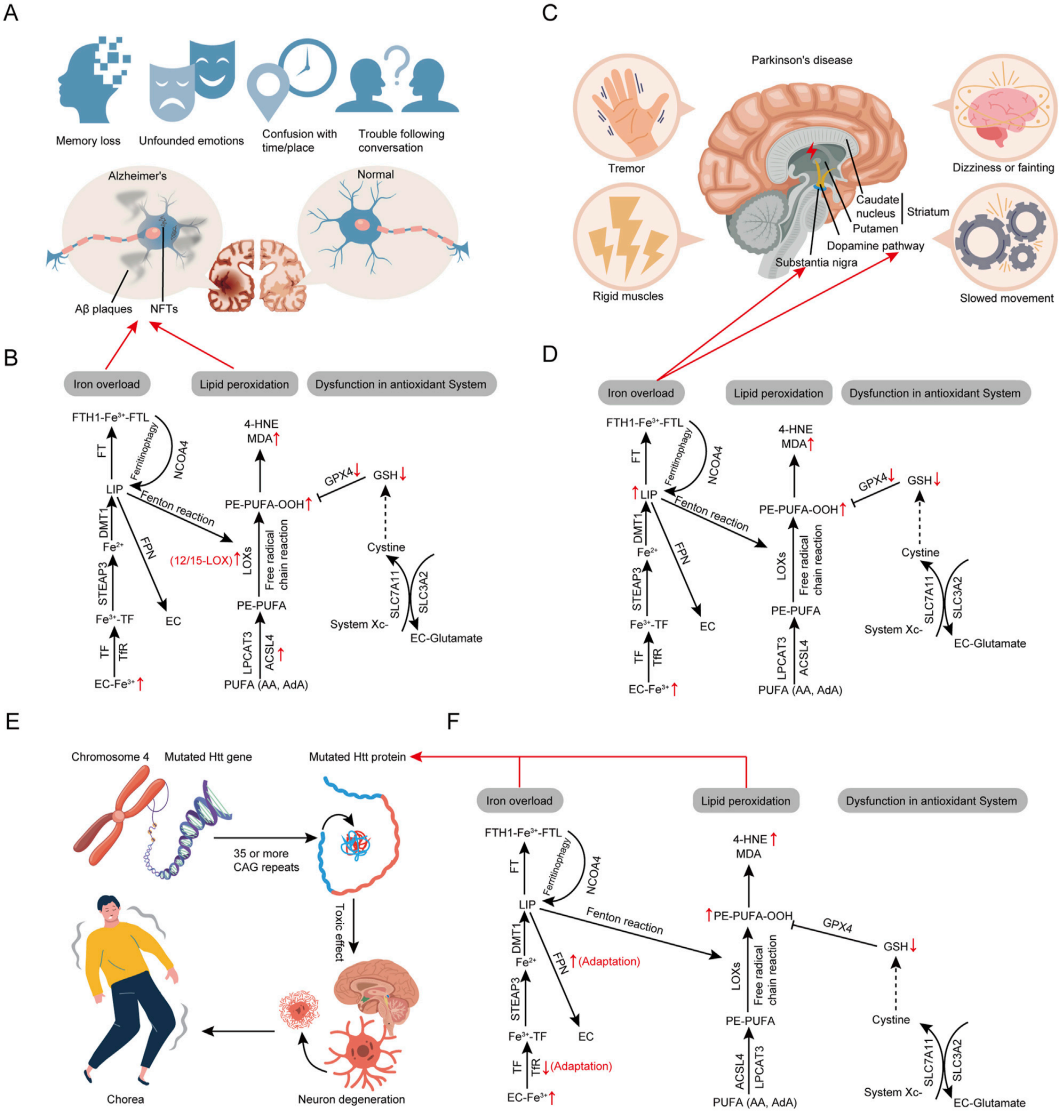
Core regulatory molecules and signaling pathways of ferroptosis in neurodegenerative diseases. **(A)** The pathogenesis of Alzheimer’s disease. **(B)** Alterations in ferroptosis pathways in Alzheimer’s disease. **(C)** The pathogenesis of Parkinson’s disease. **(D)** Alterations in ferroptosis pathways in Parkinson’s disease. **(E)** The pathogenesis of Huntington’s disease. **(F)** Alterations in ferroptosis pathways in Huntington’s disease. Third-party elements were sourced under CC BY (modifiable, commercial use with attribution). CC BY materials originate from the Freepik library (https://www.freepik.com/).

AD is the most prevalent age-related NDDs worldwide (Scheltens et al., 2021). In AD, brain regions associated with memory and cognition accumulate amyloid-beta (Aβ) plaques and neurofibrillary tangles (NFTs) formed by hyperphosphorylated Tau protein, leading to dysfunction in cortical and hippocampal neurons (Figure 4A; Khan et al., 2020). The progression of AD involves neuronal degeneration, potentially due to a combination of genetic and environmental factors. Clinical manifestations include behavioral changes, progressive memory loss, delusions, hallucinations, and decline in fine motor skills, ultimately rendering patients unable to live independently (Figure 4A; Citron, 2010). Ferroptosis is a crucial mechanism of neurodegenerative change in AD, driven primarily by intracellular iron accumulation, microglial activation, GSH metabolism dysregulation, and oxidative stress (Figure 4B; Ashraf et al., 2020; Jakaria et al., 2021).

Iron deposition has been confirmed in the cortical regions of AD patients’ brains (Tao et al., 2014), potentially linked to poor vascular conditions, aging, and neuroinflammation (Figure 4B; Nikseresht et al., 2019). Additionally, iron overload positively correlates with cognitive decline in AD patients (Wang F. et al., 2022). Disrupted iron metabolism is a significant contributing factor in AD. Elevated brain iron levels can accelerate the production of Aβ plaques, promote the hyperphosphorylation of Tau protein, and speed up the formation of NFTs, ultimately leading to neuronal dysfunction, death, and progressive loss of brain function (Figure 4B; Yamamoto et al., 2002; Liu et al., 2018). Iron contributes to ferroptosis not only through its intrinsic toxicity but also by mediating the generation of toxic lipid peroxides. Lipid peroxidation in AD patients’ brain tissues has been demonstrated, with increased activity of lipid peroxidation enzymes such as 12/15-LOX and ACSL4 and elevated levels of highly reactive secondary products like MDA detected in certain brain regions (Praticò et al., 2004; Jia et al., 2023; Rao et al., 2021). Lipid peroxidation is involved in the misfolding and degradation of Aβ proteins; inhibiting 12/15-LOX can improve phospholipid metabolism in AD rat brains, reducing Aβ/Tau protein levels (Figure 4B; Czapski et al., 2016; Giannopoulos et al., 2013; Ellis et al., 2010). The antioxidant system led by GSH/GPX4 is involved in reversing ferroptosis. Reduced GSH levels in the hippocampus and frontal cortex are linked to severe cognitive impairment, suggesting GSH as an AD biomarker (Ayton et al., 2020). Since GSH and L-cysteine cannot effectively cross the blood-brain barrier, oral supplements are ineffective. However, NAC, a precursor that can cross the barrier, regulates GSH levels, exerts neuroprotective effects, and inhibits ferroptosis in AD models (Tardiolo et al., 2018). Additionally, studies show that GPX4 inactivation induces hippocampal neuron death, while alpha-lipoic acid protects neurons by regulating GPX4 expression (Zhang YH. et al., 2018). A better understanding of the mechanisms of ferroptosis in AD could facilitate the development and application of anti-ferroptosis strategies, potentially slowing or preventing the progression of AD.

### 3.6 Mechanisms of ferroptosis in Parkinson’s disease

PD ranks as the second most common age-related NDDs worldwide (Elbaz et al., 2016), characterized clinically by resting tremors, muscle rigidity, and disturbances in gait and posture, causing significant distress to patients and their families (Figure 4C; Cacabelos, 2017). The primary pathophysiological mechanisms of PD include the deposition of alpha-synuclein (a-syn), formation of Lewy bodies, and the reduction in dopaminergic neurons, leading to a deficiency of dopamine in the nigrostriatal pathway (Figure 4C; Park et al., 2022). Dopamine is a crucial neurotransmitter, and its deficiency impedes neural transmission, thereby causing motor dysfunction (Klein et al., 2019). Currently, dopamine-based therapies such as levodopa are used to alleviate early motor symptoms of PD, but these treatments have significant side effects and do not halt disease progression (Borovac, 2016). Therefore, protecting dopaminergic neurons from damage or death remains a longstanding primary focus of PD research. Research indicates that processes such as apoptosis, necrosis, and autophagy are involved in the degenerative loss of dopaminergic neurons (Dionísio et al., 2021), yet these mechanisms do not fully elucidate the pathological processes of PD. Although the precise etiology of PD remains unclear, factors such as oxidative stress, lipid metabolism dysregulation, metal ion metabolic disorders, mitochondrial dysfunction, and glial cell activation are known to contribute to the progression of PD, suggesting that ferroptosis plays a significant role in its pathogenesis (Wang ZL. et al., 2022).

Increased iron load and exacerbated lipid peroxidation are key features of ferroptosis, aligning closely with the molecular biological changes observed in the brains of PD patients (de Farias et al., 2016; Mochizuki et al., 2020). In PD, iron levels are elevated in the substantia nigra pars compacta (SNpc) and correlate positively with disease severity, suggesting that iron, as a potent reductant, induces lipid peroxidation leading to ferroptosis (Mochizuki et al., 2020; He et al., 2020). Accumulated iron can also induce the transition of a-syn from an alpha-helical to a beta-sheet structure, a conformational feature of Lewy bodies in the substantia nigra (SN) of PD patients, potentially contributing to the onset of PD (Figure 4D; el-Agnaf and Irvine, 2002; Hallacli et al., 2022). Moreover, iron acts as a strong reductant not only generating ROS within neurons but also oxidizing dopamine (Guiney et al., 2017). Oxidative stress is recognized as a major pathogenic mechanism in PD. Studies indicate that various types of ROS could serve as biomarkers to distinguish stages of PD, with MDA being the best single marker and L-OOH activity significantly associated with advanced PD features (de Farias et al., 2016),highlighting the role of lipid peroxidation in mediating neuronal damage in PD. Additionally, studies indicate a weakening of the GSH/GPX4 antioxidant system in PD tissues. Reduced levels of GSH are observed in PD (Mandal et al., 2023),with GSH depletion considered a crucial factor in the dysfunction of dopaminergic (DA) neurons, rendering them more susceptible to oxidative damage (Smeyne and Smeyne, 2013; Bjørklund et al., 2021). Bellinger et al. demonstrated that overall GPX4 levels are significantly reduced in the substantia nigra of PD patients compared to controls, but are increased relative to the density of surviving nigral neurons (Bellinger et al., 2011). This suggests that the reduction in GPX4 may mediate ferroptosis in some nigral neurons during PD progression, while the elevated GPX4 in surviving neurons represents a protective response against oxidative stress and neurodegeneration. These studies suggest a significant link between ferroptosis and PD, indicating that targeting ferroptosis may become an important therapeutic strategy in PD management (Figure 4D).

### 3.7 Mechanisms of ferroptosis in Huntington’s disease

HD is a hereditary neurodegenerative disorder caused by autosomal dominant inheritance. Clinically, it is characterized by involuntary choreiform movements, dementia, and emotional disturbances (Figure 4E; Ross and Tabrizi, 2011). HD is induced by the expansion of CAG repeats in the Huntingtin (Htt) gene, resulting in the formation of mutant Huntingtin (mHtt) (Figure 4E; Shafie et al., 2024). Extensive research on HD has identified oxidative damage, lipid peroxidation, abnormal glutamate levels, iron accumulation, GSH dysregulation, and reduced GPX activity in both HD patients and animal models (Weiland et al., 2019; Johnson et al., 2012). The primary pathological processes involve the following: First, mHtt is cleaved at several points to generate various toxic fragments, which form monomers or small oligomers in neurons. Second, these cytotoxic fragments inhibit proteasome function and autophagy, leading to abnormal protein aggregation and mitochondrial dysfunction. Subsequently, excessive ROS, significant lipid peroxidation, and iron accumulation collectively result in ferroptosis. Additionally, oxidative stress, lipid peroxidation, and iron homeostasis imbalance exacerbate the aggregation of Htt with other proteins, leading to increased glutamate excitotoxicity, disrupted mitochondrial function, altered autophagy mechanisms, impaired axonal transport, and ultimately neuronal degeneration, thereby causing the motor, cognitive, and behavioral symptoms of HD (Reichert et al., 2020).

Excess iron accumulation is a major cause of oxidative stress and a key trigger of ferroptosis in HD (Muller and Leavitt, 2014). Magnetic resonance imaging (MRI) and quantitative susceptibility mapping (QSM) studies show increased iron levels in the occipital cortex, globus pallidus, and putamen of HD patients (Rosas et al., 2012; van Bergen et al., 2016). FT-iron levels in the striatum rise significantly, while TfR levels decrease, and FPN levels increase to manage the excess iron (Bartzokis et al., 2007; Chen et al., 2013; Simmons et al., 2007). Iron supplementation worsens neurodegeneration in HD mice by reducing striatal volume (van Bergen et al., 2016). Conversely, intraventricular administration of the iron chelator DFO improves striatal pathology and motor phenotypes in R6/2 HD mice (Chen et al., 2013). Increased lipid peroxidation is a key characteristic in HD patients (Klepac et al., 2007). 4-HNE, a secondary product of lipid peroxidation, is elevated and colocalizes with mHtt inclusions in striatal neurons of R6/2 HD mouse models (Lee et al., 2011). This elevated lipid peroxidation is also detected in corticostriatal brain slices of mN90Q73 HD mouse models, as well as in the cerebrospinal fluid of HD patients (Skouta et al., 2014; Reddy and Shirendeb, 2012). Inhibition of lipid peroxidation with Ferrostatin-1 (Fer-1) significantly improves neuropathology in R6/2 HD mouse models (Lee et al., 2011). HD patients exhibit inhibition of antioxidant systems related to ferroptosis, characterized by lower GSH levels (Klepac et al., 2007). Consistently, Kumar et al. found decreased GSH levels in the striatum, cortex, and hippocampus of 3-nitropropionic acid (3-NP)-induced HD mice (Kumar et al., 2010). Supplementation with cystamine and cysteamine reduced 3-NP-induced neuronal death and restored GSH levels in this HD model (Mao et al., 2006). Currently, there are no disease-modifying drugs available for HD; treatments primarily aim to alleviate symptoms such as motor dysfunction, cognitive deficits, and psychiatric manifestations. Overall, ferroptosis plays a crucial role in HD pathogenesis, and targeting ferroptosis represents a promising therapeutic strategy for HD (Figure 4F).

## 4 Common compounds/drugs inhibiting ferroptosis in central nervous system diseases

The studies on ferroptosis in central nervous system disorders have preliminarily demonstrated that ferroptosis inhibition holds significant potential in conditions such as IS, PD, AD, and TBI. Numerous common synthetic compounds and drugs have demonstrated ferroptosis inhibition across a wide array of disease models. Below, we outline key ferroptosis inhibitors, emphasizing their mechanisms of action (Table 1). These inhibitors act by specifically reducing free ferrous ions, enhancing antioxidant defenses, inhibiting lipid peroxidation, or indirectly inhibiting ferroptosis through other molecular pathways (Table 1).

**TABLE 1:** Overview of the main classes of ferroptosis inhibitors.

Category	Compound/drug	Mechanism	References
Iron chelators	Deferoxamine, Deferiprone, Deferasirox, Ciclopirox, 2,2-Bipyridyl, 1,10-phenant hroline, AKI-02	Reduce intracellular labile iron, inhibit the Fenton reaction	Dixon et al. (2012), Zhu et al. (2023a), Martin-Sanchez et al. (2017)
Endogenous RTAs	Vitamin E, tocotrienols, α-tocopherol, CoQ10, BH4, Vitamin K1, GSSH	Restrain LOX PUFA oxygenation	Shimada et al. (2016), Kolbrink et al. (2022b), Barayeu et al. (2023b), Li et al. (2013), Fanet et al. (2021), Mirończuk-Chodakowska et al. (2018), Zhitkovich (2019)
CoQ10 analog, Antioxidants	Idebenone	Target lipid peroxyl radicals, inhibit lipid peroxidation	Bersuker et al. (2019), Doll et al. (2019)
Synthetic RTAs, Ferrostatins	Ferrostatin-1, UAMC-3203, SRS11-92, SRS9-11, SRS16-86, UAMC-2418	Scavenge ROS, reduce labile iron in cells, inhibit lipid peroxidation	Dixon et al. (2012), Skouta et al. (2014), Zilka et al. (2017), Hofmans et al. (2016), Devisscher et al. (2018)
Synthetic RTAs, Liproxstatins	Liproxstatin-1, Liproxstatin-2	Scavenge ROS, activate Nrf2, restore GPX4, inhibit lipid peroxidation	Zilka et al. (2017), Friedmann Angeli et al. (2014), Alli et al. (2023)
Synthetic RTAs with tricyclic aromatic rings	Phenoxazines, Phenothiazine	Inhibit lipid peroxidation using tricyclic RTAs	Shah et al. (2017), Yang et al. (2021), Farmer et al. (2022), You et al. (2022)
Nitroxide-based synthetic RTAs	Tetramethylpiperidine-N-oxyl, Phenylhydroxylamine-N-oxide	Block the Fenton reaction, inhibit hydroxyl radical formation	Patanè et al. (2023), Nutting et al. (2018)
Mitochondria-targeted synthetic RTAs	XJB-5-131, JP4-039	Mitigate lipid peroxidation, target mitochondria to scavenge ROS	Krainz et al. (2016)
Phenolic synthetic RTAs	Butylated hydroxytoluene (BHT), butylated hydroxyanisole (BHA)	Scavenge free radicals, inhibit lipid peroxidation, protect membranes	Nieva-Echevarría et al. (2017), Sun et al. (2020)
Other Synthetic RTAs	SKI II, Serdemetan, AZD3463, Bazedoxifene, CuATSM, CuATSP, Necrostatin-1	Trap radicals, neutralize oxidation	Zilka et al. (2021), Conlon et al. (2021), Lum et al. (2021), Southon et al. (2020), Dennys et al. (2023), Mallais et al. (2023), Tonnus et al. (2021)
LOX inhibitors	Zileuton (A-64077), AA-861 (docebenone), PD-146176, MK-886, BWA4C	Inhibit lipoxygenases or related proteins to reduce lipid peroxidation	Zilka et al. (2017), Shah et al. (2018), Friedmann Angeli et al. (2014), Liu et al. (2015), Gregus et al. (2013), Li et al. (2014)
Inhibitors of 15LOX-2/PEBP1 complexa	FerroLOXIN-1, FerroLOXIN-2	Inhibit lipoxygenases or related proteins to reduce lipid peroxidation	Dar et al. (2023)
ACSL4 inhibitors	Troglitazone, Rosiglitazone, Pioglitazone	Inhibit ACSL4, block PUFA activation, reduce lipid peroxidation	Doll et al. (2017), Bellezza et al. (2018)
Deuterated PUFAs	RT-001	Deuterate C-D bonds, make D-PUFA resistant to lipid peroxidation	Zesiewicz et al. (2018)
GPX4 activators	PKUMDL-LC-101, PKUMDL-LC-101-D04	Activate GPX4,eliminate lipid hydroperoxides, inhibit ferroptosis	Li et al. (2019b)
Selenium supplementation, Selenoproteins	Selenium, Methylselenocysteine, Selenocystamine	Increase GPX4, boost lipid peroxide scavenging, raise selenoproteins	Alim et al. (2019), Ingold et al. (2018), Cai et al. (2017), Fei et al. (2024), Tuo et al. (2021)
Neurotransmitter	Dopamine	Increase the stability of GPX4	Wang et al. (2016b)
Targeting protein synthesis	Cycloheximide	Inhibit xCT protein synthesis	Rush et al. (2012)
Reducing agent	β-mercaptoethanol	Reduce cystine to cysteine	Sha et al. (2015)
Nrf2 activators	Bardoxolone methyl (BXM), Omaveloxolone	Activate Nrf2, inhibit ferroptosis by binding to AREs	Bellezza et al. (2018), Reisman et al. (2019), Profeta et al. (2023)
mTORC1 inhibitors	Sepanisertib (INK128), AZD8055	Inhibit mTORC1, block ferroptosis induced by class I FINs	Zhang et al. (2021f), Yi et al. (2020)
JNK and p38 inhibitors	SP600125	Inhibit JNK to suppress Erastin-induced ferroptosis	Yu et al. (2015)
p38 inhibitors	SB202190	Inhibit JNK to suppress Erastin-induced ferroptosis	Yu et al. (2015)
AMPK inhibitors	A769662, AICAR	Activate AMPK to reduce PUFA-PEs, inhibit ferroptosis	Lee et al. (2020b)
ACC1 inhibitors	5-(tetradecyloxy)-2-furoic acid	Inhibit ACC1 to reduce fatty acid synthesis	Currais et al. (2022)
PKC inhibitors	Go6983, Enzastaurin	Inhibit PKC to suppress Erastin-induced ferroptosis	Zhang et al. (2022b)
Dipeptidyl-peptidase-4 inhibitors	Vildagliptin, alogliptin, linagliptin	Reduce lipid peroxidation via inhibiting DPP4	Xie et al. (2017)

### 4.1 Inhibition of ferroptosis through iron metabolism

To address ferroptosis and iron overload-related damage, various iron chelators have been developed. Deferoxamine (DFO), Deferiprone (DFP), and Deferasirox (DFX) are currently widely used. DFO, approved by the FDA, mitigates ferroptosis by chelating Fe^3+^, reducing ROS, and upregulating GPX4, FTH1, and System Xc- (Abdul et al., 2021; Zhang Y. et al., 2020; Zeng et al., 2021). DFO has demonstrated protective effects in SCI and IS, significantly reducing infarct size and improving neurological recovery in experimental models, though its short half-life limits clinical application (Yao et al., 2019; Millán et al., 2021; Jones et al., 2022). To address this issue, oral chelators like DFP and DFX were developed. Although DFP has demonstrated nephroprotective effects in glycerol-induced acute kidney injury (AKI) models, both DFP and DFX are inevitably associated with adverse effects, including granulocyte deficiency and renal toxicity (Lecornec et al., 2022; Kattamis, 2019). Ciclopirox (CPX), initially an FDA-approved antifungal agent, has shown significant effects against ferroptosis and suppresses non-small cell lung cancer (NSCLC) growth through iron chelation (Lin et al., 2021; Lu et al., 2022). Similarly, 2,2-Bipyridyl (2,2-BP) and 1,10-Phenanthroline (1,10-PT) chelate mitochondrial iron, reducing ferroptosis and mitochondrial ROS accumulation *in vitro*, particularly in models involving zero-valent iron nanoparticles (Huang et al., 2019; Chen et al., 2020). A novel chelator, AKI-02, has exhibited significant protection in AKI models by reducing oxidative stress and ferroptosis-induced damage (Rayatpour et al., 2022).

### 4.2 Inhibition of ferroptosis through lipid metabolism

#### 4.2.1 Radical-trapping antioxidants

Radical-trapping antioxidants (RTAs) are essential for preventing ferroptosis by scavenging lipid peroxyl radicals and halting lipid peroxidation (Scarpellini et al., 2023a). Unlike the system Xc-/GSH/GPX4 pathway, which employs two-electron reductions to neutralize phospholipid hydroperoxides, RTAs use one-electron reduction mechanisms to stabilize radicals and protect cellular membranes (Maiorino et al., 2018; Zilka et al., 2017). Endogenous RTAs, naturally present in organisms, include phenolic antioxidants like Vitamin E, enzymatic systems such as CoQ10 and BH4 pathways, and sulfur-based compounds like glutathione hydropersulfide (GSSH) (Zilka et al., 2017; Pope and Dixon, 2023a). Furthermore, researchers have identified numerous other exogenous RTAs, which collectively mitigate oxidative stress and protect against ferroptosis-driven damage (Table 1).

##### 4.2.1.1 Endogenous radical-trapping antioxidants

Vitamin E integrates into cell membranes, capturing lipid peroxyl radicals via single-electron transfer, terminating chain reactions, and preserving membrane integrity. It works synergistically with selenium (Se) and selenium-dependent enzymes like GPX for antioxidant defense (Saito, 2021). Additionally, Vitamin E reduces Fe3+ within LOX-15, thereby inhibiting lipid peroxidation (Tarangelo et al., 2022). α-Tocopherol (α-TOH), the most bioactive form of vitamin E, acts as a phenolic RTA, effectively scavenging lipid peroxyl radicals to inhibit ferroptosis (Jiang et al., 2021; Shah et al., 2018). However, its efficacy is reduced due to strong hydrogen bonding between its phenolic -OH group and polar phospholipid heads (Shah et al., 2019). Tocotrienols, another vitamin E variant, have demonstrated superior inhibition of ferroptosis compared to α-TOH (Bayır et al., 2020). Additionally, Trolox, a common water-soluble vitamin E derivative, is a potent antioxidant with strong radical-scavenging activity (Kang et al., 2018).

CoQ10, through the FSP1/CoQ10 pathway, is reduced to CoQ10H2 by FSP1, using NADPH. This reduced form of CoQ10 suppresses lipid peroxidation and ferroptosis (Bersuker et al., 2019; Doll et al., 2019). The role of CoQ10 in antioxidant defense and ferroptosis inhibition was further confirmed by the discovery of ferroptosis inducer FIN56, which binds to SQS, a key enzyme in cholesterol synthesis, thereby suppressing CoQ10 (Shimada et al., 2016). Additionally, Idebenone, a synthetic CoQ10 analog requiring exogenous supplementation, mimics CoQ10 by targeting lipid peroxyl radicals (Bersuker et al., 2019; Doll et al., 2019). BH4 is a potent endogenous RTA involved in CoQ10H2 synthesis. The GCH1/DHFR/BH4 pathway exerts antioxidant effects independently of the Xc-GSH-GPX4 and NADPH-FSP1-CoQ10 axes (Akiyama et al., 2023). GCH1 serves as the rate-limiting enzyme for BH4 synthesis, while DHFR reduces BH2 to BH4 using NAD(P)H (Soula et al., 2020; Kraft et al., 2020). Overexpression of GCH1 and elevated BH4 levels enhance CoQ10H2 production, reducing ferroptosis sensitivity by depleting PUFA-PLs (Hu et al., 2022).

Vitamin K (VK), including Vitamin K1 (VK1), plays a crucial role in blood clotting, but its antioxidant properties are increasingly recognized (Mishima et al., 2023). VK1 has been shown to prevent lipid peroxidation and acts as an effective endogenous antioxidant, particularly in acute kidney injury (Kolbrink et al., 2022a). VKH2, also known as phyllohydroquinone, is the reduced form of vitamin K that neutralizes lipid peroxides and inhibits ferroptosis in GPX4-deficient models (Mishima et al., 2022a). FSP1 plays a crucial role in the reduction of vitamin K to VKH2 (Li et al., 2023a; Mishima et al., 2022b). Both VKH2 and CoQ10H2 belong to the 1,4-benzoquinone/hydroquinone antioxidant class (Li et al., 2023b).

GSSH, a specific type of hydropersulfide (RSSH), is formed from GSH with an attached -SSH group. Its RTA activity is intrinsically linked to its synthesis and cycling within the hydropersulfides/trans-sulfuration (RSSH/TSP) pathway (Barayeu et al., 2023a). GSSH operates independently of GPX4 and is more effective than vitamin E due to its lower hydrogen bond acidity, making it a potent early responder to ferroptosis induction (Barayeu et al., 2023a; Wu Z. et al., 2022; Chauvin et al., 2017).

##### 4.2.1.2 Synthetic RTAs

Synthetic RTAs are engineered molecules that mimic natural antioxidants, offering enhanced stability, potency, and bioavailability to scavenge radicals, prevent lipid peroxidation, and inhibit ferroptosis, with potential therapeutic applications in oxidative stress-related diseases like neurodegeneration and iron overload (Scarpellini et al., 2023b; Pope and Dixon, 2023b).

Ferrostatin-1 (Fer-1), identified in 2012, was the first ferroptosis inhibitor, preventing lipid hydroperoxide accumulation in HT-1080 cells in an erastin-induced model (Dixon et al., 2012). In 2017, Pratt’s group revealed Fer-1’s RTA mechanism, showing that the N-cyclohexyl moiety serves as a lipophilic anchor in membranes (Zilka et al., 2021). Additionally, both the amine group and the lipophilic anchor are critical for maintaining its activity (Skouta et al., 2014). Modification of the ethyl chain and the introduction of a benzylic moiety on the aromatic amine produced derivatives such as SRS11-92 (EC50 = 6 nM), which demonstrated significantly enhanced potency. However, substituting the ethyl ester with an amide group, as in SRS9-11 (EC50 = 950 nM), resulted in a notable reduction in activity, though this finding was later contradicted by Hofmans et al. (Skouta et al., 2014; Hofmans et al., 2016). Scouta et al. improved Fer-1’s plasma stability by replacing the ethyl ester with a tert-butyl ester and adding an imine, yielding SRS16-86 (EC50 = 350 nM) with enhanced stability but reduced activity due to weaker target binding (Linkermann et al., 2014). To improve pharmacokinetics, UAMC-2418 was synthesized by replacing the labile ester with a sulfonamide group and adding a benzyl ring to enhance stability and potency (Hofmans et al., 2016). Further modifications, including solubility-enhancing groups, led to UAMC-3203, which demonstrated superior potency, stability, and solubility, with no toxicity in mouse models (Devisscher et al., 2018). It protected against iron overload-induced multiorgan dysfunction (Van Coillie et al., 2022), improved post-resuscitation myocardial dysfunction in rats (Jin et al., 2022), and delayed relapse and disease progression in relapsing–remitting multiple sclerosis models (Van San et al., 2023).

Like Fer-1, Liproxstatin-1 (Lip-1) and Liproxstatin-2 (Lip-2) are ferroptosis inhibitors identified through small molecule screening (Friedmann Angeli et al., 2014; Alli et al., 2023). Lip-1, featuring a spiroquinoxalinamine scaffold with a critical NH group, demonstrates nanomolar potency in ferroptosis inhibition, with its quinoxaline ring playing a key role in blocking peroxyl radicals (Zilka et al., 2017). Along with UAMC-3203, Lip-1 demonstrates superior activity, solubility, and stability in mouse models (Van Coillie et al., 2022). Recently, Lip-2, an analog of Lip-1, showed improved pharmacokinetics and greater effectiveness in treating lupus nephritis both *in vitro* and *in vivo* (Alli et al., 2023).

Phenothiazines (PTZs) and phenoxazines (PNXs), as tricyclic aromatic amine-based ROS scavengers, exhibit potent antiferroptotic activity and favorable pharmacokinetics (Shah et al., 2017). In PTZs, substitutions at the C-10 position, such as alkyl or aryl groups, reduce activity, whereas modifications at the C-2 position significantly enhance potency, such as featuring a 2-phenyl-methyl piperazine at C-2, which demonstrated remarkable activity with an EC50 of 0.5 nM in erastin-induced ferroptosis in HT-1080 cells (Yang et al., 2021). PNXs exhibit even greater potency, with optimization efforts focusing on improving metabolic stability and lipophilicity (Shah et al., 2017; Farmer et al., 2022). Electron-withdrawing groups at positions C2, C3, C7, and C8, reduce activity, while electron-donating groups enhance it (Farmer et al., 2022). Non-oxidizable groups, such as CF3 or tert-butyl, at C3 or C7 improve metabolic stability, as demonstrated in mouse liver microsomes (Farmer et al., 2022).

Besides Ferrostatins, Liproxstatins, tricyclic aromatic amine-based RTAs, and other synthetic RTAs with various functional groups also inhibit ferroptosis (Table 1). Nitroxide RTAs, such as Tetramethylpiperidine-N-oxyl (TEMPO), block Fenton reactions and scavenge ·OH(403, 404). Compounds like XJB-5-131 and JP4-039 specifically target mitochondria to scavenge ROS and mitigate lipid peroxidation (Krainz et al., 2016). Phenolic RTAs, including Butylated Hydroxytoluene (BHT) and Butylated Hydroxyanisole (BHA), scavenge free radicals and inhibit lipid peroxidation through hydrogen donation (Nieva-Echevarría et al., 2017; Sun et al., 2020). Several other synthetic RTAs, including SKI II, Serdemetan, AZD3463, Bazedoxifene, CuATSM, CuATSP, and Necrostatin-1, exhibit diverse antioxidant mechanisms (Zilka et al., 2021; Conlon et al., 2021; Lum et al., 2021; Southon et al., 2020; Dennys et al., 2023; Mallais et al., 2023; Tonnus et al., 2021). FDA-approved drugs, including SKI II, Serdemetan, AZD3463, and Bazedoxifene, act as potent ferroptosis inhibitors by capturing free radicals and chelating iron in HT-1080 cell models (Conlon et al., 2021). Copper-based complexes, such as CuATSM and its derivative CuATSP, demonstrate enhanced activity through iron chelation and mitochondrial protection, with CuATSP showing superior permeability and ferroptosis inhibition (Lum et al., 2021; Southon et al., 2020; Dennys et al., 2023). Additionally, multifunctional compounds like Necrostatin-1 not only inhibit necroptosis but also trap free radicals and generate sulfenic acid intermediates to suppress ferroptosis (Mallais et al., 2023; Tonnus et al., 2021). These diverse mechanisms highlight promising therapeutic strategies for ferroptosis-related diseases.

#### 4.2.2 LOX inhibitors

LOXs drive ferroptosis by catalyzing PUFA oxidation, making them promising therapeutic targets for mitigating ferroptosis-related diseases (Shah et al., 2018). Among LOX inhibitors, Zileuton (A-64077), a LOX-5 inhibitor, reduces oxidative stress by regulating ROS levels in retinal pigment epithelial (RPE) cells, offering potential for retinal disease treatment (Liu et al., 2015; Lee et al., 2022). AA-861 (Docebenone), targeting LOX-5/12, effectively suppress lipid peroxidation and mitigate ferroptosis-related damage (Scarpellini et al., 2023a). Other LOX-5 inhibitors, such as PD-146176 (Walters et al., 2018), MK-886 (Shi KN. et al., 2023), and BWA4C (Franchi-Miller and Saffar, 1995), have also demonstrated strong inhibitory effects in preclinical studies. Anthonymuthu et al. revealed that inhibiting the 15LOX-2/PEBP1 complex, even without directly targeting 15-LOX, can reduce the production of 15-hydroperoxy-eicosatetraenoyl phosphatidylethanolamine (15-HpETE-PE), thereby effectively suppressing lipid peroxidation and ferroptosis (Anthonymuthu et al., 2021). FerroLOXIN-1 and FerroLOXIN-2, developed by Dar et al., inhibit lipid peroxidation and ferroptosis both *in vitro* and *in vivo* by specifically targeting the 15LOX-2/PEBP1 complex, which has become a critical target in ferroptosis research (Dar et al., 2023).

#### 4.2.3 ACSL4 inhibitors

Glitazones, including TRO, ROSI, and PIO, mitigate ferroptosis by targeting ACSL4, thereby preventing PUFA activation and reducing lipid peroxidation (Kim et al., 2001). These TZDs, often considered PPARγ activators and insulin-sensitizing agents, also specifically inhibit ACSL4; however, their antioxidant properties suggest that the inhibition of ferroptosis may partly result from off-target effects (Xu et al., 2022; Parker, 2002; Garg et al., 1979). In response to this challenge, Doll et al. demonstrated that in ACSL4 knockout MEF cells, Rosiglitazone, Pioglitazone, and Troglitazone prevent RSL3-induced membrane lipid peroxidation and ferroptosis, significantly extending the survival of ACSL4 knockout mice (Doll et al., 2017; Bellezza et al., 2018).

#### 4.2.4 Deuterated PUFAs

Deuterated PUFAs (D-PUFAs), by incorporating C-D bonds, enhance resistance to lipid peroxidation, thereby preventing ferroptosis. This mechanism effectively inhibits lipid peroxidation and demonstrates significant protective effects, especially in neurodegenerative disease models (Navratil et al., 2018; Shchepinov, 2020). Based on this principle, Retrotope developed RT-001 (containing deuterated linoleic acid) and initiated its clinical trials to further verify its potential in treating related diseases (Brenna et al., 2020; Zesiewicz et al., 2018).

### 4.3 Inhibition of ferroptosis through antioxidation

Ferroptosis is an iron-dependent cell death driven by the accumulation of lipid peroxides due to impaired antioxidant systems (Wang S. et al., 2024). The three key axes in ferroptosis defense are: the System Xc^−^/GSH/GPX4 axis, where System Xc^−^ imports cystine for GSH synthesis, enabling GPX4 to reduce lipid peroxidation (Chen et al., 2021b); the FSP1/CoQ10/NADPH axis, where FSP1 reduces CoQ10 to inhibit lipid peroxidation (Bebber and von Karstedt, 2023) (Bentinger et al., 2007); and the GCH1/BH4/DHFR axis, which generates BH4 to neutralize free radicals and prevent lipid peroxidation (Ma T. et al., 2022).

Activating GPX4 is a promising strategy to control lipid peroxidation, but designing activators is challenging. Li et al. designed eight allosteric GPX4 activators with a unique mechanism, distinct from typical ferroptosis inhibitors (Li C. et al., 2019). PKUMDL-LC-101 and its optimized analog PKUMDL-LC-101-D04 were the most effective in boosting GPX4 activity, though their IC50 values are above 100 μM, making them moderate ferroptosis inhibitors. This strategy may be combined with RTAs for enhanced efficacy (Li C. et al., 2019). The main antioxidants associated with GPX4, GSH and its precursor NAC, play a crucial role in inhibiting ferroptosis by enabling GSH to reduce lipid hydroperoxides to non-toxic lipid alcohols (Maiorino et al., 2018).

Several compounds target pathways that indirectly enhance antioxidant defenses by promoting GPX4 activity or GSH synthesis, thereby suppressing lipid peroxidation and inhibiting ferroptosis. For instance, Selenium supplementation and selenoproteins enhance GPX4 activity, boost lipid peroxide scavenging, and increase selenoproteins, reducing lipid peroxidation (Alim et al., 2019; Ingold et al., 2018; Cai et al., 2017; Fei et al., 2024; Tuo et al., 2021). Dopamine, a neurotransmitter, stabilizes GPX4, further inhibiting ferroptosis (Wang D. et al., 2016). Cycloheximide inhibits protein synthesis by binding to the 60S ribosomal subunit in eukaryotic cells, blocking peptide chain elongation (Schneider-Poetsch et al., 2010). In ferroptosis research, it inhibits xCT protein synthesis, reducing cystine uptake, limiting GSH synthesis, weakening antioxidant defenses, and promoting ferroptosis (Rush et al., 2012). β-Mercaptoethanol, a potent reducing agent, converts cystine in the culture medium into cysteine, enhancing cellular cysteine uptake, GSH synthesis, and antioxidant capacity (Sha et al., 2015). These approaches, by modulating System Xc^−^/GSH/GPX4 pathways, provide effective strategies for controlling ferroptosis.

Additionally, Nrf2, a master regulator of antioxidant responses, enhances the expression of key genes involved in antioxidant defense (such as SLC7A11, GPX4, and HO-1) and GSH synthesis, playing a pivotal role in maintaining redox balance and suppressing ferroptosis (Yan et al., 2023). Bardoxolone methyl (BXM) activates the p62/Keap1/Nrf2 pathway, promoting Nrf2 activation. The activated Nrf2 binds to AREs, protecting cells from ferroptosis (Bellezza et al., 2018; Reisman et al., 2019). Omaveloxolone, a synthetic oleanolic acid derivative, activates the Nrf2 pathway to protect cells from ferroptosis and shows potential in treating mitochondrial dysfunction-related conditions (Pilotto et al., 2024). It is currently approved for the treatment of Friedreich’s ataxia, a genetic disorder characterized by mitochondrial impairment (Pilotto et al., 2024; Profeta et al., 2023).

### 4.4 Other ferroptosis inhibitors

Some small molecule inhibitors target pathways intersecting with ferroptosis regulation by modulating specific mechanisms (Table 1). For example, mTORC1 inhibitors like Sepanisertib (INK128) and AZD8055 inhibit mTORC1 and block ferroptosis induced by class I ferroptosis inducers (FINs) (Zhang et al., 2021f; Yi et al., 2020). JNK and p38 inhibitors, such as SP600125 and SB202190, suppress the MAPK pathway, which contributes to ferroptosis under specific conditions (Yu et al., 2015). AMPK activators, including A769662 and AICAR, reduce polyunsaturated fatty acid-containing phosphatidylethanolamines (PUFA-PEs), limiting substrates for lipid peroxidation (Lee H. et al., 2020). Acetyl-CoA carboxylase 1 (ACC1) inhibitors like 5-(tetradecyloxy)-2-furoic acid inhibit ACC1 to decrease fatty acid synthesis (Currais et al., 2022). Protein kinase C (PKC) inhibitors, such as Go6983 and Enzastaurin, reducing oxidative stress and lipid peroxidation linked to ferroptosis (Zhang HL. et al., 2022). Dipeptidyl-peptidase-4 (DPP4) inhibitors like Vildagliptin, Alogliptin, and Linagliptin reduce lipid peroxidation by inhibiting DPP4, which regulates glucose metabolism and redox reactions, affecting cellular oxidative stress responses (Xie et al., 2017). These small molecules modulate ferroptosis indirectly by influencing key metabolic and cellular pathways.

### 4.5 Clinical trials of ferroptosis inhibition therapies for central nervous system diseases

Several antiferroptotic therapeutics have advanced to clinical trials, offering hope for treating ferroptosis-related conditions, especially in CNS diseases, where ferroptosis plays a key role in neuronal damage and progression. We summarize the completed clinical trials and those terminated due to low efficacy or severe side effects for these therapies in CNS diseases, as shown in Table 2.

**TABLE 2:** Clinical trials with anti-ferroptosis therapeutics.

Mechanism	Molecule	Pathological population	Phase/design	Outcome	Trial ID/Ref
Iron chelator	Deferoxamine (intravenous)	IS	Phase II, randomized, double-blind, placebo-controlled	Nonsignificant improvement: DFO at 40–60 mg/kg/day reduced transferrin saturation (TSAT) by 30%–40% at 72 h. At day 90, 50%–60% of patients treated with DFO achieved a favorable outcome (mRS ≤2), compared to 31% in the placebo group (P = 0.10)	NCT00777140/(Millán et al., 2021)
Iron chelator	Deferoxamine mesylate (intravenous)	ICH	Phase II, randomized, double-blind, placebo-controlled	Nonsignificant improvement: At day 90, 34% of patients in the DFO mesylate (32 mg/kg/day for 3 consecutive days) group achieved a favorable outcome (mRS ≤2) compared to 33% in the placebo group (P = 0.82). Serious adverse events occurred in 27% of the DFO group and 33% of the placebo group (P = 0.65)	NCT02175225/(Selim et al., 2019)
Iron chelator	Deferoxamine (intramuscular)	AD	Single-blind, placebo-controlled	Significant improvement: DFO (125 mg twice daily for 5 days/week over 24 weeks) significantly slowed the rate of decline in daily living skills in AD patients compared to placebo (P = 0.028)	Unregistered/(Crapper McLachlan et al., 1991)
Iron chelator	Deferiprone (oral)	AD	Phase II, randomized, double-blind, placebo-controlled	Worsening: DFP (15 mg/kg twice daily for 12 months) accelerated cognitive decline compared to placebo (cognitive score reduction: DFP −0.80 vs. placebo −0.30, P = 0.002). DFP reduced hippocampal iron (P = 0.03) but did not prevent hippocampal volume loss (P = 0.61). Neutropenia incidence was higher in the DFP group (7.5%) compared to similar studies (1.6%–4.4%)	NCT03234686/(Ayton et al., 2024)
Iron chelator	Deferiprone (oral)	PD	Phase II, randomized, double-blind, placebo-controlled	Worsening: DFP (15 mg/kg twice daily for 36 weeks) accelerated symptom progression in PD (MDS-UPDRS increase: DFP +15.6 vs. placebo +6.3, P < 0.001). It reduced substantia nigra iron (P < 0.001) but did not improve dopamine transporter density. SAEs (9.7% vs. 4.8%) included 2 cases of agranulocytosis and 3 of neutropenia in the DFP group	NCT02655315/(Devos et al., 2022)
Iron chelator	Deferiprone (oral)	PD (dopaminergic therapy)	Phase II, randomized, double-blind, placebo-controlled	Nonsignificant improvement: DFP (20 or 30 mg/kg/day for 6 months) significantly reduced iron in the dentate nucleus (P < 0.001) and caudate nucleus (20 mg/kg/day: P = 0.007; 30 mg/kg/day: P = 0.0002), but minimally affected the substantia nigra (P = 0.20). In the 30 mg/kg/day group, UPDRS III improved from 10.57 ± 1.34 to 8.5, and PDQ-39 scores slightly decreased from 24.28 ± 6.29 to ∼22 (P > 0.05). Deferiprone was well tolerated; 2 patients developed neutropenia, with mild muscle/joint pain and gastrointestinal upset in a few cases	NCT01539837/(Martin-Bastida et al., 2017)
RTA, GSH increase	GSH (intranasally)	PD	Phase I, randomized, double-blind, placebo-controlled	Nonsignificant improvement: GSH (300 or 600 mg/day, 3 doses daily for 3 months) reduced total UPDRS scores: −5.3 (300 mg), −4.3 (600 mg) vs. +1.1 (placebo; P = 0.09). UPDRS Part III improved by 3.1 (300 mg) and 1.4 (600 mg), vs. worsened 0.8 (placebo; P = 0.15). GSH was well tolerated, with mild nasal irritation; one patient withdrew due to side effects	NCT01398748/(Mischley et al., 2015)
RTA, GSH increase	GSH (intravenous)	PD (dopaminergic therapy)	Pilot study, randomized, double-blind, placebo-controlled	Nonsignificant improvement: GSH (1400 mg, 3 times weekly for 4 weeks) showed a mean improvement of 2.8 points in UPDRS motor scores at week 4 compared to placebo (P = 0.32). At the 8-week follow-up, the GSH group worsened by 3.5 points from baseline, while the placebo group improved by 2 points (P = 0.54). GSH was well tolerated with no SAEs	Unregistered/(Hauser et al., 2009)
RTA, GSH increase	NAc (oral)	PD	Phase I + II randomized, quadruple-blind, placebo-controlled	Nonsignificant improvement: At Week 4, the NAC 1800 mg/day group showed a mean reduction of 7.23 points in PDQLQ scores compared to baseline, while the NAC 3600 mg/day group showed a reduction of 6.71 points. In contrast, the placebo group exhibited only a minimal reduction of 0.40 points	NCT01470027/-
RTA, GSH increase	NAc (intravenous + oral)	PD	Phase (NA) randomized open-label	Significant improvement: NAC (50 mg/kg intravenous weekly +600 mg oral twice daily for 3 months) significantly increased DAT binding in the caudate (0.15, P = 0.014) and putamen (0.12, P = 0.039). It also reduced total UPDRS scores by 4.29 points (P < 0.001), including motor (−2.88, P = 0.003) and non-motor (−1.41, P = 0.01) improvements. NAC was well tolerated, with no SAEs	NCT02445651/(Monti et al., 2016; Monti et al., 2019)
RTA, GSH increase	NAc (oral)	PD (dopaminergic therapy)	Phase II, open-label	Worsening: NAC (6000 mg/day for 4 weeks) increased UPDRS scores in PD patients from 32.6 to 36.6. Brain GSH levels showed no significant change (+6%, P = 0.3 at 7T; +10%, P = 0.06 at 3T). Blood antioxidant markers, such as GSH/GSSG ratios, increased significantly (P < 0.05), while lipid peroxidation markers (4-HNE and MDA) remained unchanged. Mild to moderate adverse events, including increased drooling and tremor, were reported in 3 patients, with 1 withdrawing early	NCT02212678/(Coles et al., 2018)
RTA, CoQ10 increase	CoQ10 (oral)	HD	Phase III, randomized, double-blind, placebo-controlled	Worsening: Oral CoQ10 (up to 2400 mg/day for up to 5 years) showed no significant difference from placebo in Total Functional Capacity (TFC) score changes or survival time (P = 0.76). Secondary outcomes showed no meaningful benefits, except for a marginal improvement in Stroop word-reading scores (3.88, 95% CI 0.31–7.44, P = 0.03). The trial was terminated early due to futility, with CoQ failing to slow functional decline in HD. A slightly higher mortality rate was observed in the CoQ group (7.3% vs. 4.2%)	NCT00608881/(McGarry et al., 2017)
RTA, CoQ10 increase	CoQ10 (oral)	PD	Phase III, randomized, double-blind, placebo-controlled	Worsening: Oral CoQ10 (1200 or 2400 mg/day combined with 1200 IU/day vitamin E for up to 16 months) increased UPDRS total scores: 6.9 points (placebo), 7.5 points (1200 mg/day, P = 0.49), and 8.0 points (2400 mg/day, P = 0.21). No significant benefits were observed in secondary outcomes, and the trial was terminated early due to futility. CoQ10 was generally well tolerated, with mild gastrointestinal discomfort and insomnia reported	NCT00740714/(Beal et al., 2014)
ACSL4 inhibitors	Rosiglitazone XR (oral)	AD	Phase II, randomized, double-blind, placebo-controlled	Nonsignificant improvement: Oral RSG-XR (4 mg/day for 1 month, 8 mg/day for 11 months) modestly improved CMRglu after 12 months (treatment: −6.3%, placebo: −13.1%; difference: 6.8%, P = 0.17). Brain atrophy rates showed no significant difference (P = 0.22), and ADAS-Cog scores worsened more in the treatment group (+7.62) compared to placebo (+5.44), with a difference of 2.18 points (P = 0.26)	NCT00265148/(Tzimopoulou et al., 2010)
ACSL4 inhibitors	Rosiglitazone XR (oral)	AD	Phase III, randomized, double-blind, placebo-controlled	Nonsignificant improvement: In a Phase III, randomized, double-blind, placebo-controlled trial, 1,468 mild-to-moderate AD patients received rosiglitazone extended-release (RSG XR) as an adjunct to acetylcholinesterase inhibitors (AChEI) for 54 weeks. ADAS-Cog scores changed by −0.15 (RSG XR) vs. −0.14 (placebo; P = 0.95), and ADCS-ADL scores decreased by 0.5 (RSG XR) vs. 0.6 (placebo; P = 0.88)	NCT00348140/-
ACSL4 inhibitors	Pioglitazone (oral)	IS/TIA	Phase III, randomized, double-blind, placebo-controlled	Significant Improvement: Oral Pioglitazone (45 mg/day for up to 5 years) significantly reduced the risk of stroke or myocardial infarction (MI) in insulin-resistant patients, with a HR of 0.76 (95% CI: 0.62–0.93, P = 0.007). No significant differences were observed in all-cause mortality or cognitive outcomes. Adverse events included increased risks of weight gain (52.2% vs. 33.7%), edema (35.6% vs. 24.9%), and bone fractures requiring hospitalization (5.1% vs. 3.2%). Pioglitazone reduced the risk of ischemic stroke by 28% (HR = 0.72, P = 0.005), with no effect on hemorrhagic stroke (HR = 1.00, P = 1.00)	NCT00091949/(Kernan et al., 2016)
ACSL4 inhibitors	Pioglitazone (oral)	AD	Phase III, randomized, double-blind, placebo-controlled	Nonsignificant Improvement: Pioglitazone (0.8 mg/day for up to 5 years) did not significantly delay the onset of mild cognitive impairment (MCI) due to Alzheimer’s disease (2.7% vs. 3.3%, HR = 0.80, 99% CI: 0.45–1.40, P = 0.307). Secondary outcomes, including cognitive composite scores and ADCS-ADL assessments, showed no meaningful benefits. Adverse events included bone fractures (6.5% vs. 7.4%) and cardiac disorders (0.1% vs. 0.5%), with no significant safety concerns	NCT01931566/(Burns et al., 2021)

Several investigational therapies targeting iron metabolism and antioxidant activity show promise in inhibiting ferroptosis. Among these, iron chelators such as DFO and DFP are the most extensively studied in clinical settings (Devos et al., 2020); however, their therapeutic use is hindered by off-target effects and the essential role of iron in homeostasis (Table 2). DFO, which does not cross the blood-brain barrier, has been tested in IS, ICH, and AD (Millán et al., 2021; Farr and Xiong, 2021). In IS, intravenous DFO at 40–60 mg/kg/day showed a trend toward improved functional outcomes at day 90, with 50%–60% of patients achieving a favorable outcome (modified Rankin Scale [mRS] ≤2) compared to 31% in the placebo group (P = 0.10) (Millán et al., 2021). Similarly, in ICH, DFO demonstrated minimal improvement (34% favorable outcomes vs. 33% with placebo), with no significant difference (P = 0.82) and a comparable rate of serious adverse events (SAEs) between groups (27% vs. 33%, P = 0.65) (Selim et al., 2019). In AD, intramuscular DFO significantly slowed the rate of dementia progression and decline in daily living skills (P = 0.028), despite its limited CNS penetration and an unclear mechanism (Crapper McLachlan et al., 1991).

DFP, in contrast, can cross the blood-brain barrier and selectively reduce iron in overloaded CNS regions, making it a focal point in studies on neurodegenerative diseases such as PD (Cabantchik et al., 2013; Mahoney-Sánchez et al., 2021; Negida et al., 2024; Ayton et al., 2024). In PD, DFP selectively reduced iron levels in degenerated regions, as confirmed by MRI, without affecting healthy CNS areas (Devos et al., 2014; Devos et al., 2022). Pilot studies (NCT01539837) observed a nonsignificant trend toward improved motor function and quality of life with DFP at 30 mg/kg/day when combined with dopaminergic therapy, with significant reductions in iron levels in the dentate and caudate nuclei (P < 0.001), and good tolerability (Martin-Bastida et al., 2017). However, a larger trial in *de novo* patients without dopaminergic therapy (NCT02655315) found that DFP at 15 mg/kg twice daily for 36 weeks significantly worsened motor symptoms, with Movement Disorder Society-Unified Parkinson’s Disease Rating Scale (MDS-UPDRS) scores increasing more in the DFP group compared to placebo (DFP: +15.6 vs. placebo: +6.3, P < 0.001) (Devos et al., 2022). This adverse effect likely stems from interference with dopamine synthesis, as DFP reduces iron availability for tyrosine hydroxylase, a key enzyme. SAEs, including agranulocytosis and neutropenia (9.7%), were also more frequent in this trial, highlighting the need to consider patient characteristics, particularly concurrent dopaminergic therapy, when evaluating DFP in PD. DFP has also been evaluated in other neurodegenerative diseases. In AD, oral DFP at 15 mg/kg twice daily reduced hippocampal iron (P = 0.03) but paradoxically accelerated cognitive decline, likely due to disruptions in critical iron-dependent processes (Ayton et al., 2024).

In addition to therapies targeting iron, various lipid peroxidation inhibitors have undergone clinical trials. General antioxidants, some of which exhibit ferroptosis inhibition *in vitro*, have also been evaluated clinically but with limited efficacy (Table 2). In a Phase II trial (NCT01398748) on PD, intranasal GSH at doses of 300 mg/day and 600 mg/day showed nonsignificant trends toward motor improvement, with slight reductions in total and motor Unified Parkinson’s Disease Rating Scale (UPDRS) scores compared to placebo (total UPDRS: −5.3 and −4.3 vs. +1.1; P = 0.09) (Mischley et al., 2015). Similarly, an intravenous GSH trial found no significant differences in motor or activities of daily living (ADL) scores between the GSH and placebo groups at week 4 or during follow-up, suggesting only minimal symptomatic benefits (Hauser et al., 2009). These results highlight the challenges of using GSH therapeutically in PD, despite its essential role as a GPX4 cofactor.

NAC, a precursor for GSH synthesis, has shown mixed results in clinical trials for PD. An oral NAC trial (NCT01470027) showed no significant improvement in UPDRS scores, while another trial (NCT02212678) reported worsening UPDRS scores with no change in brain GSH levels, despite improvements in antioxidant markers (Coles et al., 2018). Conversely, a study combining intravenous and oral NAC (NCT02445651) reported significant motor (P = 0.003) and non-motor (P = 0.01) UPDRS improvements in 42 PD patients who continued standard dopaminergic therapy. Enhanced dopamine transporter (DAT) binding was also observed in the caudate (P = 0.014) and putamen (P = 0.039) (Monti et al., 2016; Monti et al., 2019). These findings highlight the potential influence of delivery method, dosing strategy, and concurrent dopaminergic therapy on NAC’s efficacy. CoQ10, another exogenous antioxidant reduced by FSP1, has shown limited clinical efficacy in NDDs.In a Phase III trial (NCT00740714), oral CoQ10 at 1200 or 2400 mg/day, combined with 1200 IU/day of vitamin E, did not slow PD progression, as UPDRS scores increased similarly across all groups (Beal et al., 2014). In HD, a Phase II trial (NCT00608881) was terminated early due to futility, as CoQ10 failed to slow functional decline or improve survival, with a slightly higher, though not statistically significant, mortality rate observed in the treatment group (McGarry et al., 2017). These findings highlight the challenges of translating CoQ10’s theoretical neuroprotective effects into meaningful clinical outcomes.

ACSL4 facilitates lipid peroxidation in ferroptosis by incorporating PUFAs into PLs, and its inhibition suppresses ferroptosis, offering potential therapeutic benefits in diseases linked to oxidative stress and iron accumulation. Rosiglitazone, an oral ACSL4 inhibitor, has been evaluated in clinical trials for AD, but none demonstrated significant clinical benefits. A Phase III trial (NCT00348140) with rosiglitazone XR (extended-release) showed identical results, offering no measurable benefit in AD. Similarly, another Phase II trial (NCT00265148) investigating rosiglitazone XR observed a modest improvement in Cerebral metabolic rate of glucose (CMRglu) over 12 months (treatment: −6.3%, placebo: −13.1%; difference: 6.8%, P = 0.17), but brain atrophy rates and cognitive decline showed no significant differences (Tzimopoulou et al., 2010). Cognitive decline in the treatment group exceeded the placebo group by 2.18 points (P = 0.26) on Alzheimer’s Disease Assessment Scale-Cognitive Subscale (ADAS-Cog). These findings suggest that, despite minor metabolic improvements, the XR formulation of rosiglitazone is ineffective in halting disease progression in Alzheimer’s disease.

Pioglitazone, an ACSL4 inhibitor, showed significant cardiovascular benefits in a Phase III trial (NCT00091949) involving insulin-resistant patients with a history of IS or transient ischemic attack (TIA). The trial reported a 24% reduction in the risk of stroke or myocardial infarction (hazard ratio [HR] = 0.76, 95% confidence interval [CI]: 0.62–0.93, P = 0.007) and a 50% reduction in diabetes incidence (3.8% vs. 7.7%, HR = 0.48, P < 0.001). However, cognitive outcomes and all-cause mortality showed no significant differences. Adverse events included increased risks of weight gain, edema, and fractures requiring hospitalization (Kernan et al., 2016). In a separate Phase III (NCT01931566), pioglitazone (0.8 mg/day) did not significantly delay the onset of mild cognitive impairment due to AD (2.7% incidence with pioglitazone vs 3.3% with placebo; HR = 0.80, 99% CI: 0.45–1.40; P = 0.307). Secondary outcomes, including cognitive composite scores and Alzheimer’s Disease Cooperative Study–Activities of Daily Living (ADCS-ADL) assessments, showed no meaningful benefits (Burns et al., 2021). Considering the evidence, pioglitazone may reduce cardiovascular events in insulin-resistant patients with a history of IS or TIA; however, it does not delay the onset of mild cognitive impairment due to AD.

Given the limited success of clinical trials involving ferroptosis inhibitors like DFO, DFP, and CoQ10 in treating CNS diseases such as AD, PD, and IS, there is a pressing need for alternative therapeutic strategies. Recent research has highlighted the potential of natural products in modulating ferroptosis pathways within CNS disorders. These naturally occurring compounds may offer novel avenues for intervention, warranting further investigation into their mechanisms and efficacy.

## 5 Natural flavonoids as ferroptosis inhibitors for the therapy of CNS diseases

**FIGURE 5:**
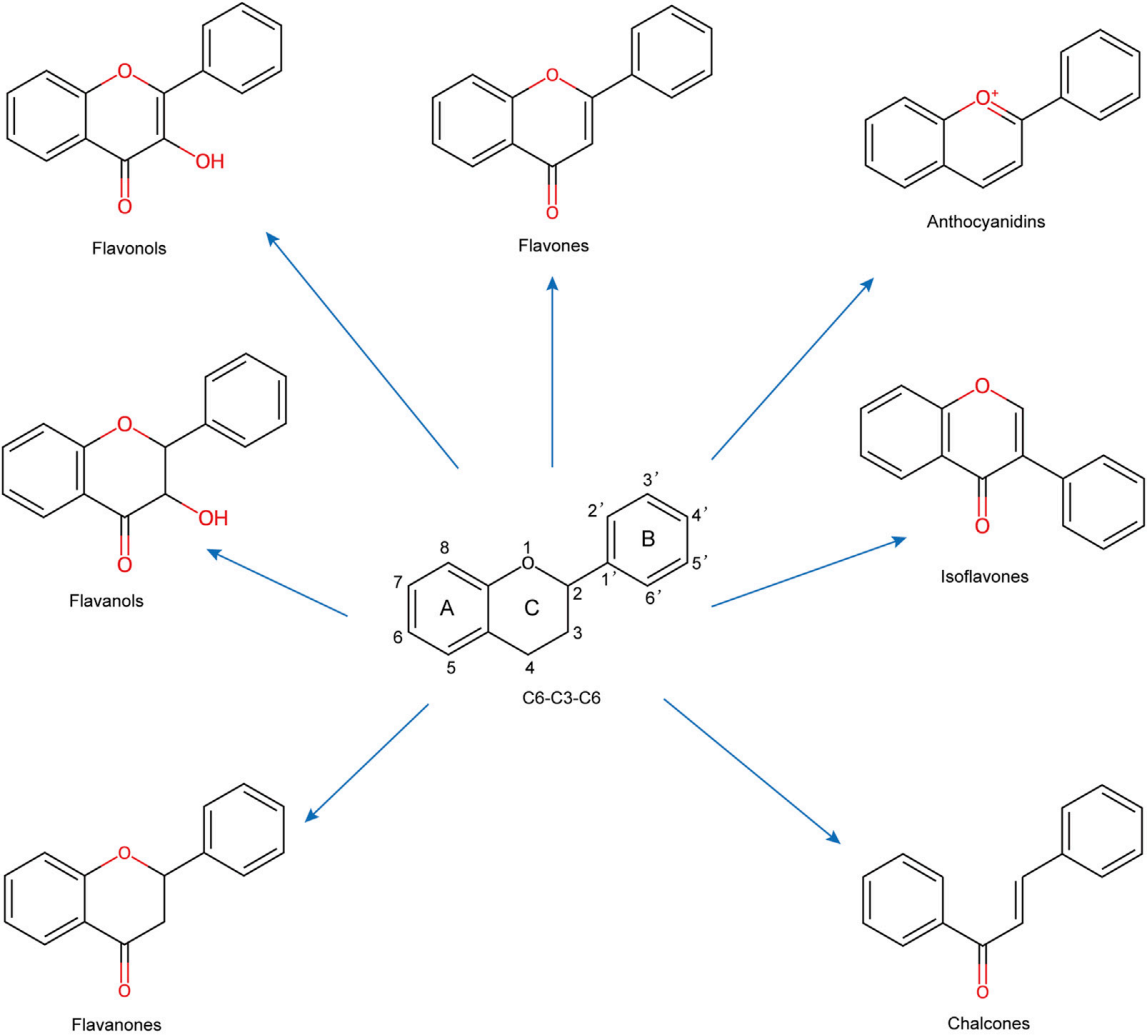
Structures of skeletons from seven flavonoid subclasses: flavones, flavonols, flavanones, flavanols, isoflavones, anthocyanidins, and chalcones.

Flavonoids are one of the largest classes of plant polyphenols, characterized by a C6-C3-C6 backbone consisting of three rings labeled as A, B, and C (Dias et al., 2021). Based on their chemical structure, flavonoids are divided into seven subclasses: flavones, flavonols, flavanones, flavanols, isoflavones, anthocyanidins, and chalcones (Figure 5; Shen et al., 2022; Živanović et al., 2024). These categories differ in C-ring oxidation, substitution patterns, and functional groups, which influence their biological activities (Dias et al., 2021). Flavonoids, with antioxidant, anti-inflammatory, and protective effects, have been shown to inhibit ferroptosis driven by iron metabolism and lipid peroxidation (Bellavite, 2023; Shen et al., 2022; Liga et al., 2023; Zhou et al., 2023). We utilized multiple electronic databases, including PubMed, Web of Science, Scopus, Medline, and CNKI, to review studies on various natural flavonoids as ferroptosis inhibitors for treating central nervous system diseases. This study focused on basic research, prioritizing the efficacy and mechanisms of natural products validated through robust animal models. The goal is to provide new therapeutic strategies for CNS diseases by targeting ferroptosis pathways with these natural products.

### 5.1 Flavones

**FIGURE 6:**
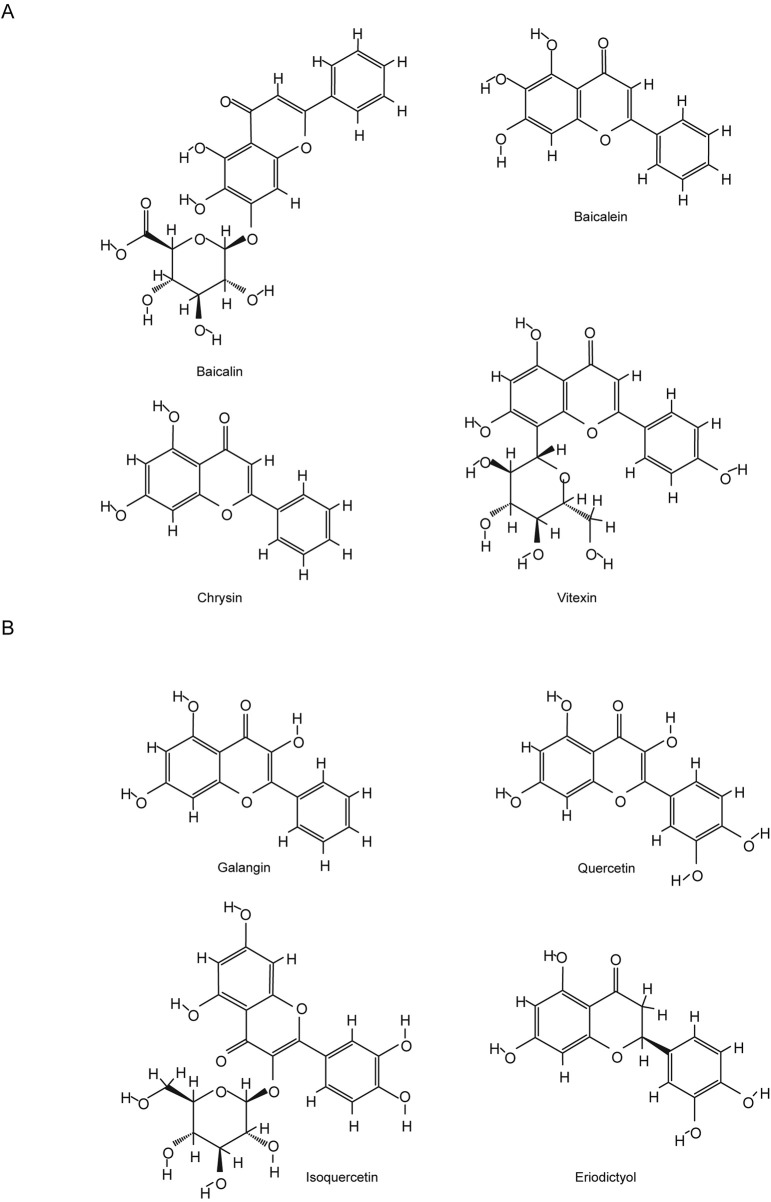
Structures of anti‐ferroptosis flavonoids in central nervous system diseases. **(A)** Flavone structures. **(B)** Flavonol and flavanone structures.

Flavones, one of the major flavonoid subgroups, are characterized by a 2-phenylchromen-4-one backbone, distinguished by the absence of a hydroxyl group at position C3 (Figure 5; Shen et al., 2022). These flavone compounds, including acacetin, baicalein, baicalin, chrysin, vitexin, scutellarein, kumatakenin, and apigenin-7-O-(6″-p-coumaroyl)-glucoside, prevent and treat diseases by targeting multiple pathways to inhibit ferroptosis (Živanović et al., 2024). For instance, acacetin combats liver lipid accumulation in non-alcoholic fatty liver disease in mice by suppressing endoplasmic reticulum stress-induced ferroptosis (Jiang et al., 2023). Apigenin-7-O-(6″-p-coumaroyl)-glucoside reduces ischemia/reperfusion injury in mice by lowering ROS and Fe2+ levels and inhibiting HO-1 (Feng et al., 2022). Kumatakenin limits iron accumulation and lipid peroxidation in DSS-induced acute colitis in mice, while luteolin decreases ROS, MDA, and iron levels, enhancing GPX4 protein levels in heart ischemia/reperfusion models (Arenbaoligao et al., 2023; Wang IC. et al., 2023). Scutellarein modulates the GPX4 antioxidant system to thwart ferroptosis in chronic obstructive pulmonary disease induced by LPS and cigarette smoke in mice (Liu L. et al., 2023). It is noteworthy that baicalein, baicalin, chrysin, and vitexin have been shown in preclinical animal models to treat CNS diseases by inhibiting ferroptosis (Table 3), with their chemical structures illustrated in Figure 6A. Baicalin and baicalein are the two flavones most extensively studied in in vivo experiments related to CNS diseases.

**TABLE 3:** Natural flavones as ferroptosis inhibitors in central nervous system diseases.

Natural plant compounds	Disease	*In vivo* model	Pharmacological intervention/Harvest	Ferroptosis inhibition mechanism	Ref
Baicalin (BC)	SAH-EBI	PCC-ABI model, SD rat	BC (100 mg/kg, ip) at 2 and 12 h post-SAH	Inhibit autophagy-dependent FT degradation; reduce Fe^2+^, MDA, ROS; upregulate GPX4, GSH	Zheng et al. (2021)
Baicalin (BC)	ICH	Collagenase IV-induced ICH model, C57BL/6 mouse	BC (20 mg/kg, gavage) at 2 h post-ICH, daily for 3 days	Upregulate GPX4 and SLC7A11; downregulate SLC11A2 and iron transport	Duan et al. (2021)
Baicalin (BC)	IS	MCAO model, mouse	Data unavailable	Upregulate MiR-556-3p, target and downregulate ACSL4	Dai et al. (2024)
Baicalin (BC)	CIRI	tBCCAO model, C57BL/6 mouse	BC (50 mg/kg, gavage), daily for 7 days	Inhibit PGE2/COX-2; downregulate DMT1; upregulate GPX4	Xiren et al. (2023)
Baicalein (BL)	TBI	CCI model, C57BL/6 mouse	BL (50 mg/kg, ip) at 10–15 min post-CCI	Reduce 15-LOX, ACSL4; increase GSH	Kenny et al. (2019)
Baicalein (BL)	TBI-PTE	FeCl3-induced post-traumatic epilepsy (PTE) model, C57BL/6 mouse	BL (50/100 mg/kg, ip) 30 min prior to FeCl3 administration	Reduce 12/15-LOX; decrease ROS, 4-HNE, PTGS2; upregulate GPX4	Li et al. (2019a)
Baicalein (BL)	CIRI	tMCAO model, C57BL/6 mouse	BL (10/80 mg/kg, ip), daily for 7 days	Upregulate GPX4, ACSL3; downregulate ACSL4	Li et al. (2022c)
Chrysin (CHY)	CIRI	tMCAO model, SD rat	CHY (50/100/200 mg/kg, gavage), twice daily for 5 days	Upregulate SLC7A11, GPX4; downregulate TFR1, PTGS2, ACSL4	Shang et al. (2023a)
Chrysin (CHY)	CIRI	tMCAO model, SD rat	CHY (50 mg/kg, gavage), twice daily for 5 days	Inhibit HIF-1α; downregulate ACSL4; upregulate GPX4, SLC7A11	Shang et al. (2023b), Shang et al. (2024)
Vitexin (VTX)	CIRI	Middle cerebral artery occlusion/reperfusion (MCAO/R) model, SD rat	VTX (45 mg/kg, ip), daily for 7 days	Target Nrf2/Keap1/HO-1 pathway	Guo and Shi (2023)

#### 5.1.1 Baicalin and baicalein

*Scutellaria baicalensis, also known as Chinese or Baikal skullcap, is a perennial herb in the Lamiaceae family* (Yin et al., 2021; Chanchal et al., 2023). Renowned for its antipyretic, anti-inflammatory, and antioxidative properties, it contains various bioactive compounds, primarily natural flavonoids like baicalin (BC) and baicalein (BL) (Ahmadi et al., 2022; Si et al., 2025). BC, a glucoside derivative of BL, is enzymatically hydrolyzed by intestinal enzymes during digestion, releasing BL, which then contributes to the pharmacological effects of both compounds (Si et al., 2025; Liu X-y et al., 2024). Studies have demonstrated that BC functions as a ferroptosis inhibitor, offering neuroprotection in CNS injury-related conditions, particularly in ischemic and hemorrhagic stroke (Duan et al., 2021; Xie et al., 2016; Zheng et al., 2021; Dai et al., 2024; Xiren et al., 2023). In an animal study, two pre-surgery intraperitoneal injections of BC (100 mg/kg) significantly improved neurological function and reduced brain water content in SAH rats undergoing preoptic cistern autologous blood injection (PCC-ABI). This was achieved by inhibiting autophagy-dependent FT degradation, reducing Fe^2+^ levels, and decreasing lipid peroxidation products (MDA and ROS) (Zheng et al., 2021). Duan et al. demonstrated that BC enhances cell viability and inhibits ferroptosis in PC12 cells treated with hemin, erastin, and RSL3 *in vitro*. *In vivo*, in a type IV collagenase-induced ICH mouse model, oral BC administration (20 mg/kg) for three consecutive days alleviated motor deficits and brain injury, reduced iron deposition in perihematomal brain tissue, and exerted anti-ferroptotic effects by upregulating the expression of GPX4 and SLC7A11 (227). Additionally, in IS, BC inhibits ferroptosis by upregulating miR-556-3p, which suppresses ACSL4 expression and reduces lipid peroxidation (Dai et al., 2024). Furthermore, Deng et al. demonstrated that BC (50 mg/kg) reduces DMT1 levels in the hippocampus of a transient bilateral common carotid artery occlusion (tBCCAO) mouse model. It also decreases prostaglandin-endoperoxide synthase 2 (PTGS2, also known as COX-2) and MDA levels, promotes iron storage, activates ferroptosis defense pathways, and ultimately improves cognitive function in CIRI (Xiren et al., 2023).

BL, a flavone from plants, offers therapeutic potential in microbial infections, cancer, neurodegenerative, and cardiovascular diseases by modulating oxidative stress, inflammation, and cell death, with strong neuroprotective effects (Paul et al., 2024). Recent studies highlight BL’s potential as a ferroptosis inhibitor in CNS diseases. It prevents erastin-induced ferroptosis by inhibiting GSH and GPX4 degradation, reducing lipid peroxidation, and activating the Nrf2 pathway to mitigate oxidative damage, showing promise in treating AD and PD (Li Y. et al., 2017). In a CCI model of TBI, a single intraperitoneal injection of BL (50 mg/kg) inhibits 15-LOX, reduces the accumulation of oxidized phosphatidylethanolamine, alleviates neuronal damage, and improves spatial memory acquisition in mice (Kenny et al., 2019). Additionally, BL significantly alleviates ferric chloride (FeCl_3_)-induced seizures in mice and reduces ferric ammonium citrate (FAC)-induced damage in HT22 hippocampal neurons by decreasing lipid peroxidation products like 4-HNE, inhibiting 12/15-LOX expression, and upregulating key ferroptosis regulators such as GPX4, thereby inhibiting ferroptosis in TBI-induced post-traumatic epilepsy (PTE) (Li Q. et al., 2019). Li et al. found that BL at 80 mg/kg mitigates neuronal death and improves cognitive function in a tMCAO mouse model. *In vitro*, BL increases the expression of GPX4, FTH1, mitochondrial ferritin (FTMT), and SLC7A11 (a key subunit of system Xc-), and ACSL3, while decreasing the expression of ACSL4 and total iron levels in OGD/R-treated HT22 cells, with effects being dose-dependent (Li M. et al., 2022). These findings indicate that baicalein reduces CIRI by lowering iron content, inhibiting lipid peroxidation, enhancing endogenous antioxidant activity, and regulating various ferroptosis-related proteins (Ming, 2022). These findings suggest that baicalein alleviates CIRI by reducing iron accumulation, inhibiting lipid peroxidation, enhancing antioxidant activity, and modulating ferroptosis-related proteins.

#### 5.1.2 Chrysin

Chrysin (CHY), a flavone found in propolis, *S. baicalensis*, and *Pterocarpus indicus*, exerts neuroprotective effects by modulating oxidative stress, inflammation, and apoptosis (Naz et al., 2019; Mishra et al., 2021). CHY protects against CIRI in both *in vivo* and *in vitro* models. In the tMCAO rat model, CHY improved neurological function and reduced infarct size by upregulating SLC7A11 and GPX4, and downregulating ferroptosis markers TfR1, PTGS2, and ACSL4, thereby lowering total iron, lipid peroxidation, and MDA levels, and mitigating oxidative stress. Prussian blue staining revealed reduced iron accumulation in brain tissue. In the erlotinib-treated HT22 hippocampal neuron model, CHY enhanced GPX4 expression, reduced ACSL4, suppressed ROS, and improved cell viability, further supporting its role in ferroptosis regulation (Shang JF. et al., 2023). Additionally, studies suggest that CHY inhibits hypoxia-inducible factor 1α (HIF-1α), which may underlie its regulation of ferroptosis-related molecules (SLC7A11, GPX4, TfR1, ACSL4, and PTGS2) (Shang J. et al., 2023; Shang et al., 2024).

#### 5.1.3 Vitexin

Vitexin (VTX), a flavone glycoside from *Vitex agnus-castus*, *Passiflora incarnata*, and *Morus alba*, displays anti-inflammatory and antioxidant properties, offering protection in oxidative stress-related diseases like seizures, cerebral ischemia, and neurotoxicity through ROS scavenging and signaling modulation (Hajdú et al., 2007; Babaei et al., 2020; Ganesan and Xu, 2017). Guo et al. demonstrated that VTX alleviated CIRI by modulating the Keap1/Nrf2/HO-1 pathway. In a rat MCAO/R model, 45 mg/kg of Vitexin administered daily via intraperitoneal injection for 7 days reduced brain injury, improved mitochondrial function, and inhibited ROS production, thereby protecting against ferroptosis. In primary cortical neurons subjected to OGD/R, Vitexin enhanced Nrf2 nuclear translocation by regulating Keap1, upregulated HO-1, GPX4, and SLC7A11, and reduced TFR1 expression (Guo and Shi, 2023).

### 5.2 Flavonols

Flavonols, also known as 3-hydroxyflavones, are characterized by a 2-phenylchromen-4-one backbone with a hydroxyl group at the C3 position. The A-ring typically carries hydroxyl groups at the 5 and 7 positions, making flavonols enriched in 3-OH groups (Shen et al., 2022). Flavonols such as kaempferol, quercetin, isorhamnetin, isoquercetin, rutin, and galangin, have demonstrated bioactive properties in inhibiting ferroptosis in both *in vitro* and *in vivo* experiments (Živanović et al., 2024). Quercetin, Galangin, and Isoquercetin, with chemical structures shown in Figure 6B, inhibit ferroptosis, thereby delaying CNS disease progression (Table 4). Kaempferol activates the Nrf2/SLC7A11/GPX4 pathway, boosting antioxidant defenses and reducing neuronal ferroptosis in OGD/R-induced damage, showing promise for CIRI treatment, though further animal studies are needed (Yuan et al., 2021).

**TABLE 4:** Natural flavonoid-based ferroptosis inhibitors in central nervous system diseases beyond flavones.

Classification	Natural plant compounds	Disease	*In vivo* model	Pharmacological intervention/Harvest	Ferroptosis inhibition mechanism	Ref
Flavonols	Galangin (GAL)	CIRI	Bilateral common carotid artery occlusion/reperfusion (BCCAO/R) model, gerbils	GAL (25/50/100 mg/kg, gavage), daily for 14 days	Activate SLC7A11/GPX4 axis; increase GSH	Guan et al. (2021)
	Quercetin (QCT)	SCI	Impact-induced SCI model, C57BL/6 mice	QCT (20 mg/kg, ip), daily for 7 days	Upregulates expression of GPX4 and PGS2, and downregulates expression of TF and Id2	Wang et al. (2023d)
	Quercetin (QCT)	PD	MPTP-induced PD model, C57BL/6 mice	QCT (60 mg/kg, ip), daily for 8 days	Activates Nrf2/GPX4/SLC7A11 signaling pathway	Lin et al. (2022)
	Isoquercetin (Iso)	CIRI	MCAO/R model, SD rats	Iso (5, 10, 20 mg/kg, gavage), daily for 3 days	Decreases the production of ROS and MDA, increases the activity of SOD and CAT, and inhibits the NOX4/ROS/NF-κB pathway by induction of Nrf2 nuclear translocation	Dai et al. (2018)
Flavanones	Eriodictyol (ERD)	AD	APPswe/PSEN1dE9 (APP/PS1) double transgene mice model	ERD (50 mg/kg, ip), three times weekly for 3 months	Target Nrf2/Keap1/HO-1; upregulate GPX4; downregulate ACSL4, TFR1	Li et al. (2022d)
Flavanols	Proanthocyanidin (PAC)	CIRI	MCAO/R model, ICR mice	PAC (25/50/100 mg/kg, gavage), daily for 7 days	Activates Nrf2/HO-1 signaling pathway	Chen et al. (2023)
	Proanthocyanidin (PAC)	SCI	Laminectomy combined with T10 spinal cord compression-induced SCI model, C57BL/6 mice	PAC (5/10 mg/kg, ip), daily for 10 days	Decreased levels of iron, TBARS, downregulation of ACSL4 and ALOX15B, upregulation of GPX4, Nrf2, and HO-1, and increased level of GSH	Zhou et al. (2020b)
	(−)-epigalocatechin-3-gallate (EGCG)	SCI	T9 spinal cord transection induced SCI model, rats	EGCG (50 μM, ip), daily for 7 days	Upregulates expression of GPX4 and FTH1,downregulates expression of ACSL4 and COX2	Wang et al. (2020b)
	Dihydromyricetin (DHM)	CIRI	MCAO/R model, SD rat	DHM (150/200/250 mg/kg, gavage), daily for 7 days	Inhibit SPHK1/mTOR; upregulate GPX4; downregulate ACSL4, PEBP1	Xie et al. (2022)
	Dihydromyricetin (DHM)	ICH	Collagenase-induced ICH model, C57BL/6 mouse	DHM (50/100/300 mg/kg, gavage), daily for 7 days	Inhibit LCN2/SLC3A2 pathway; upregulate GPX4, GSH	Liu et al. (2023b)
Isoflavones	Soybean Isoflavones (SI)	CIRI	MCAO/R model, SD rat	SI (120 mg/kg, gavage), daily for 21 days	Upregulate GPX4; reduce Fe^2+^, MDA, MPO, TNF-α, IL-1β	Li et al. (2023c)
	Calycosin (CAL)	CIRI	Transient middle cerebral artery occlusion/reperfusion (tMCAO/R) model, SD mouse	CAL (5/10/20 mg/kg, ip), daily for 8 days	Inhibit ACSL4; upregulate GPX4; reduce Fe^2+^, MDA	Liu et al. (2023c)
	Icariside II (IC II)	IS	MCAO model, C57BL/6 mouse	IC II (5/10/20 mg/kg, gavage), daily for 7 days	Target Nrf2/Keap1/HO-1, OXPHOS/NF-κB/ferroptosis pathway	Gao et al. (2023)
Chalcones	Safflower Yellow (SY), also called Carthamin Yellow (CY)	CIRI	MCAO model, SD rat	CY (20/40 mg/kg, gavage), daily for 14 days	Upregulate GPX4, GSH; downregulate ACSL4; reduce Fe^2+^, MDA, ROS; inhibit NF-κB/NLRP3	Guo et al. (2021)

#### 5.2.1 Galangin

Galangin (GAL) is an important natural flavonol primarily found in galangal and propolis, with various biological activities such as anti-inflammation, antibacterial, antioxidant, anti-aging, anti-fibrosis, and antihypertensive effects (Zhang et al., 2023; Wang D. et al., 2023). GAL alleviates CIRI in gerbil brains by inhibiting ferroptosis via the SLC7A11/GPX4 axis. In the bilateral common carotid artery occlusion/reperfusion (BCCAO/R) model, GAL treatment improved learning and memory in the Morris water maze (MWM) test, reduced lipid peroxidation, and upregulated SLC7A11 and GPX4 expression. The protective effects were diminished upon SLC7A11 knockout, indicating that GAL mitigates ferroptosis and neuronal cell death by enhancing the SLC7A11/GPX4 pathway (Guan et al., 2021). Additionally, GAL exerts antioxidant and neuroprotective effects by activating the Keap1/Nrf2/HO-1 pathway. In a 6-hydroxydopamine (6-OHDA)-induced PD model, it reduces ROS levels and activates antioxidant pathways to inhibit ferroptosis, protecting dopaminergic neurons (Chen QX. et al., 2022). These findings suggest that GAL has potential therapeutic applications in treating CIRI and PD.

#### 5.2.2 Quercetin and isoquercetin

Quercetin (QCT), a plant pigment and potent antioxidant flavonol, predominantly found in onions, grapes, berries, cherries, broccoli, and citrus fruits, serves as a versatile antioxidant known for its protective capacity against tissue damage caused by various drug toxicities (Anand David et al., 2016). QCT is employed in neuroprotective research in various animal models. For an impact-induced SCI model in C57BL/6 mice, QCT was administered intraperitoneally at 20 mg/kg daily for 7 days, which significantly reduced injury area and improved post-SCI BBB scores (Wang Y. et al., 2023). This treatment notably modulated the expression of Id2 and TF, while upregulating GPX4 and PTGS2, highlighting its protective mechanisms. Concurrently, in an MPTP-induced PD model, QCT was administered at 60 mg/kg daily for 8 days, activating the Nrf2/GPX4/SLC7A11 signaling pathway, which improved motor function and preserved dopaminergic neurons (Lin et al., 2022). Remarkably, Nrf2 knockdown reduced QCT’s protective effects against ferroptosis, emphasizing its crucial role in QCT’s neuroprotective actions.

Isoquercetin (Iso), or quercetin-3-O-β-D-glucopyranoside, differs from quercetin by the addition of a glucose molecule at its 3-hydroxyl group. Found in various plants, fruits, and vegetables, Iso exhibits higher bioavailability and offers protection against oxidative stress, cancer, cardiovascular diseases, diabetes, and allergies in both *in vivo* and *in vitro* studies (Valentová et al., 2014). Iso has been explored for its neuroprotective effects in CIRI using an MCAO/R model in Sprague-Dawley rats. Administered via gavage at a minimum dose of 5 mg/kg daily for 3 days, Iso significantly reduced infarct volume and brain water content. Furthermore, Iso treatment ameliorated neurological deficits, as indicated by lower neurological severity scores. Mechanistically, Iso reduced ROS and MDA levels, enhanced SOD and CAT activity, and suppressed the NOX4/ROS/NF-κB pathway by promoting Nrf2 nuclear translocation (Dai et al., 2018).

### 5.3 Flavanones

Flavanones, also called dihydroflavonoids, are defined by a saturated C-ring resulting from the lack of a double bond between C2 and C3. These compounds are predominantly found in citrus fruits, such as oranges and lemons, with hesperidin and naringin as representative examples (Shen et al., 2022). Recent studies reveal that flavanones hesperidin, naringenin, naringin, eriodictyol, pinocembrin, and kumatakenin possess ferroptosis-inhibitory properties. Both hesperidin and pinocembrin activate the Nrf2 pathway, showing therapeutic potential in intervertebral disc degeneration models (Zhu J. et al., 2023; Wang H. et al., 2023). Naringenin and naringin, through activation of the Nrf2/HO-1 signaling pathway, exhibit protective effects in AgNPs-induced lung injury in ICR mice and a streptozotocin-induced diabetic cardiac autonomic neuropathy model in Sprague-Dawley rats, respectively (Zhang X. et al., 2022; Tang et al., 2022). Furthermore, kumatakenin prevents iron accumulation and lipid peroxidation, offering therapeutic benefits in acute colitis (Arenbaoligao et al., 2023).

Eriodictyol (ERD) is a flavanone found in citrus fruits, known for its anti-inflammatory, anti-cancer, neurotrophic, and antioxidant effects, with its chemical structure illustrated in Figure 6B (Deng et al., 2020; Islam et al., 2020; Lee et al., 2015). Numerous studies have demonstrated that ERD exerts its antioxidant effects through Nrf2 activation (Lee et al., 2015; Johnson et al., 2009). ERD improves cognitive function and reduces AD-related pathology in the APP/PS1 transgenic mouse model by modulating the Nrf2/Keap1/HO-1 pathway. Administered at 50 mg/kg, three times weekly for 3 months, ERD upregulated GPX4 and downregulated ACSL4 and TfR1, reducing oxidative stress and iron accumulation. In Aβ1-42-induced HT-22 cells, ERD (2, 4, and 8 μM) significantly enhanced cell survival, reduced Tau hyperphosphorylation and neurotoxicity, and decreased lipid ROS and iron levels, highlighting its neuroprotective potential (Li L. et al., 2022). These results support ERD as a promising candidate for AD treatment (Table 4).

### 5.4 Flavanols

**FIGURE 7:**
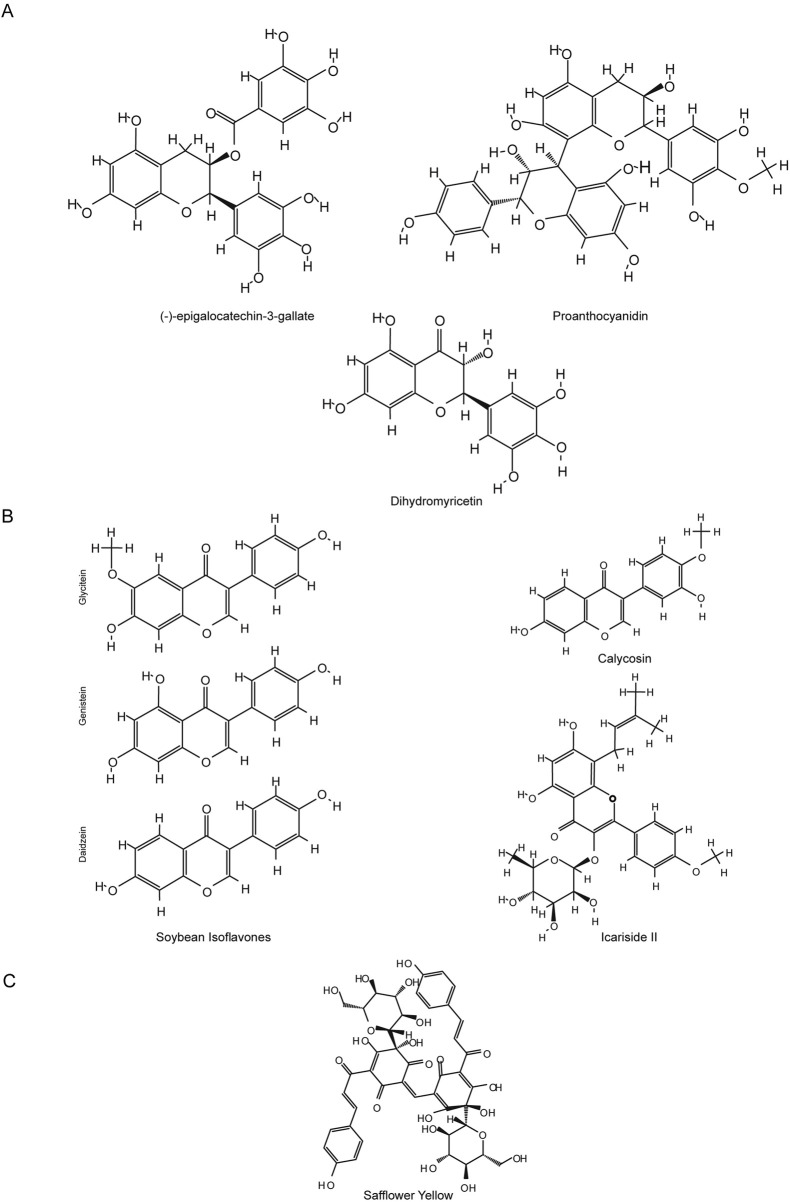
Structures of anti-ferroptosis flavonoids in CNS diseases. **(A)** Flavanol structures. **(B)** Soybean Isoflavone structures. **(C)** Chalcone structures.

Flavanols (Flavan-3-ols) are defined by a hydroxyl group at the C3 position, absence of a C2-C3 double bond, and lack of a C4 keto group. Their structural variations arise from different hydroxylation patterns on the A, B, and C rings (Shen et al., 2022). Flavanols, including catechin, epicatechin, and their derivatives, exist as monomers and form the building blocks of proanthocyanidins, their oligomeric and polymeric counterparts (Verma et al., 2024). Proanthocyanidins, (−)-epigallocatechin-3-gallate, and dihydromyricetin, representative flavanols (Figure 7A), have been shown to exert neuroprotective effects by inhibiting ferroptosis (Table 4).

#### 5.4.1 Proanthocyanidins

Proanthocyanidins (PACs) are predominantly found in berries and fruits, making them one of the richest natural sources. Edible berries such as lingonberries, cranberries, black elderberries, black chokeberries, black currants, and blueberries are particularly abundant in proanthocyanidins (Rauf et al., 2019). PACs demonstrated neuroprotective effects in models of CIRI and SCI (Table 4). In an MCAO/R model using ICR mice, prophylactic administration of PACs via gavage at a minimum dose of 25 mg/kg daily for 7 days enhanced neurological function, reduced infarct volume, and activated the Nrf2/HO-1 signaling pathway. PACs also promoted GPX4 and SLC7A11 expression, suppressed TFR1 expression, decreased Fe^2+^ levels, and mitigated lipid peroxidation, effectively inhibiting ferroptosis (Chen et al., 2023). In a T10 compression-induced SCI model in C57BL/6 mice, intraperitoneal injection of PACs at 5 or 10 mg/kg significantly reduced iron, and ACSL4 levels while increasing GSH, GPX4, Nrf2, and HO-1 expression. These molecular changes correlated with improved motor function in SCI mice, suggesting that PACs may serve as a potential therapeutic agent for spinal cord repair by inhibiting ferroptosis (Zhou H. et al., 2020).

#### 5.4.2 (−)-Epigalocatechin-3-gallate

(−)-Epigallocatechin-3-gallate (EGCG), the primary catechin in green tea (Camellia sinensis), belongs to the flavanols class and is a key active polyphenol. It has been extensively studied as a promising candidate for treating chronic inflammation and oxidative damage-related diseases (Min et al., 2015). EGCG, administered via intraperitoneal injection daily for 7 days, has been shown to exert neuroprotective effects in a T9 spinal cord transection-induced SCI model in rats. This treatment promotes functional recovery in rats following complete spinal cord transection, significantly upregulating the expression of GPX4 and FTH1, while downregulating ACSL4 and COX2 expression, suggesting its potential to modulate anti-ferroptosis pathways involved in SCI recovery (Wang J. et al., 2020).

#### 5.4.3 Dihydromyricetin

Dihydromyricetin (DHM), the main flavonoid in rattan tea, exhibits broad pharmacological effects, including cardioprotection, anti-diabetes, hepatoprotection, neuroprotection, and anti-tumor activity. Its mechanisms likely involve anti-oxidative and anti-inflammatory pathways, mediated by key molecules like AMPK, MAPK, Akt, NF-κB, and Nrf2 (Zhang J. et al., 2018). DHM inhibited the SPHK1/mTOR pathway, reducing ferroptosis and CIRI in MCAO/R rats. At doses of 150–250 mg/kg, DHM improved neurological function, reduced brain edema, and infarct size in a dose-dependent manner. In OGD/R-treated HT-22 cells, DHM lowered ROS and intracellular iron, while upregulating GPX4 and downregulating ACSL4 (Xie et al., 2022). In addition, *in vitro* studies show that DHM exerts ferroptosis-inhibiting effects in OGD/R-induced HT22 cells by activating the Nrf2/HO-1 pathway (Zhang Q. et al., 2021). In a mouse model of ICH, DHM (50–300 mg/kg, oral gavage, daily for 7 days) inhibited LCN2 to regulate SLC3A2, thereby affecting system Xc- function and suppressing ferroptosis (Liu X. et al., 2023).

### 5.5 Isoflavones

Isoflavones are chemical compounds with a 3-phenylchromen-4-one backbone. They are primarily found in legumes, particularly soybeans and their derivatives, as well as in alfalfa and chickpeas (Ku et al., 2020). Isoflavones have a structure like animal estrogens, such as estradiol-17β, and exhibit affinity for estrogen receptors, classifying them as phytoestrogens with both estrogenic and anti-estrogenic effects (Sakthivelu et al., 2008). Preclinical studies indicate that Daidzein, Biochanin A, Formononetin, and Tectorigenin inhibit ferroptosis and have been applied in APAP-induced hepatotoxicity, knee osteoarthritis, chronic kidney disease, and ureteral obstruction. Specifically, Soybean Isoflavones, Calycosin, and Icariside II exert neuroprotective effects in IS through ferroptosis inhibition (Figure 7B; Table 4).

#### 5.5.1 Soybean Isoflavones

Soybean Isoflavones (SI), plant compounds found in soybeans, belong to the isoflavonoid class of compounds. They exhibit estrogen-like properties, which play a key role in managing menopausal symptoms, osteoporosis, and cardiovascular health. While their neuroprotective effects are primarily attributed to their estrogenic activity, they also offer neuroprotection through their antioxidant properties (Kim, 2022; Qian et al., 2012; Tan et al., 2023). Research showed that administering 120 mg/kg of SI via gavage for 21 days in an MCAO/R rat model reduced Fe^2+^ and MDA levels in the ischemic penumbra, while increasing GSH and GPX4, suggesting a neuroprotective effect through antioxidant modulation and ferroptosis inhibition (Li S. et al., 2023).

#### 5.5.2 Calycosin

Calycosin (CAL) belongs to the isoflavones class and is a phytoestrogen extracted from the root of *Astragalus membranaceus*. It is widely known for its estrogen-like activity and exhibits a broad spectrum of pharmacological effects, including anticancer, anti-inflammatory, anti-osteoporotic, neuroprotective, and hepatoprotective properties (Deng et al., 2021; Gao et al., 2014). The CAL treatment group in the tMCAO/R-induced CIRI rat models significantly reduced brain edema and BBB disruption, inhibited ACSL4, upregulated GPX4, and decreased Fe^2+^ and MDA levels. In OGD/R-treated PC12 cells, CAL (60 μM for 24 h) enhanced cell survival, reduced apoptosis (as shown by Annexin V-PI staining), and decreased lipid ROS, iron accumulation, and oxidative stress markers, ultimately improving cell viability (Liu H. et al., 2023).

#### 5.5.3 Icariside II

Icariside II (IC II), a natural isoflavone from Epimedium species, exhibits potent antioxidant activity and efficacy in treating oxidative stress-induced tissue damage (Liu X. et al., 2020). IC II has demonstrated significant neuroprotective effects in ischemic stroke (IS). In the MCAO model in C57BL/6 mice, 10 mg/kg IC II alleviated neuronal damage and improved learning and memory in the Morris water maze test. In primary astrocyte OGD/R experiments, IC II (6.25 μM) enhanced Nrf2 transcriptional activity, promoted its nuclear translocation, and activated the OXPHOS/NF-κB/ferroptosis axis. It reduced cell damage by upregulating SLC7A11 and GPX4 expression while decreasing lipid peroxidation (Gao et al., 2023). Fan et al. also found that IC II activates the Keap1/Nrf2/GPX4 pathway to inhibit ferroptosis, reduce oxidative stress, and protect neural cells (Fan and Zhou, 2023). In an MPP + -induced PD model using the human neuroblastoma SK-N-SH cell line, IC II improved cell survival and reduced oxidative stress, suggesting its potential for treating both brain ischemia and neurodegenerative diseases.

### 5.6 Chalcones

Chalcones (1,3-diaryl-2-propen-1-one) are natural open-chain flavonoids characterized by up to three modified or unmodified C5-, C10-, and C15-isoprenyl groups on the A and B rings. These bioactive compounds are widely distributed in legumes, Moraceae, Zingiberaceae, and Cannabaceae (Elkanzi et al., 2022). Chalcones exhibit diverse pharmacological activities, including antioxidant, antibacterial, anti-ulcer, antiviral, antiprotozoal, and anticancer effects (Ouyang et al., 2021). As precursors to flavonoids and isoflavonoids, chalcones play a crucial role in their biosynthesis. Isoliquiritigenin, Licochalcone A, and Cardamonin exhibit anti-ferroptosis bioactive effects in various disease models. Isoliquiritigenin reduces MDA, Fe2+, and NO levels, while increasing GPX4 and Xct- expression and decreasing NCOA4 expression in an LPS-induced acute kidney injury model in C57BL/6 mice (Tang Y. et al., 2021). Licochalcone A upregulates GPX4, downregulates ACSL4, and inhibits the Nrf2/HO-1 pathway in a cardiac ischemia/reperfusion model in SD rats (Lin et al., 2023). Cardamonin enhances the expression of SLC7A11, GPX4, and p53, and reduces iNOS and COX2 levels in an osteoarthritis model in SD rats (Gong et al., 2023). These findings underscore the diverse bioactive effects of these compounds, all of which activate GPX4/GSH-related antioxidant pathways.

Safflower Yellow (SY) is a class of natural yellow pigment extracted from safflower (Carthamus tinctorius) petals (Figure 7C; Do et al., 2024). The main components include Hydroxysafflor Yellow A and Safflower Yellow B, which possess an open C-ring structure, characteristic of chalcones (Chen G. et al., 2022). SY exhibits various biological activities, such as antioxidant, anti-inflammatory, blood circulation-promoting, and cardiovascular-protective effects (Chen Y. et al., 2022; Fu et al., 2021). Although it is commonly referred to as Carthamin Yellow, this term is incorrect, as Carthamin refers to the red pigment found in safflower, while Safflower Yellow is the correct name for the yellow pigment (Savcenco, 2022). However, we have also used Carthamin Yellow (CY) as a keyword in related searches to ensure comprehensive literature retrieval. In a study by Guo et al. (Guo et al., 2021)., CY treatment for 2 weeks in an MCAO rat model improved neurological function, reduced brain water content, and diminished infarct size by inhibiting the accumulation of ferrous ions and ROS, and reversing the expression levels of ACSL4, TfR1, GPX4, and FTH1 in CIRI (Table 4). Studies have shown that Hydroxysafflor Yellow A protects PC12 cells from OGD/R injury by inhibiting ferroptosis, offering new strategies for treating degenerative diseases such as cerebral ischemia, though this has yet to be validated in animal models (Chen G. et al., 2022).

### 5.7 Anthocyanidins

Anthocyanidins are plant pigments responsible for the red, purple, and blue coloration of flowers and fruits, typically found in glycosylated forms (Cruz et al., 2022; Pan et al., 2019). They are glycosides of polyhydroxy and polymethoxy derivatives with an unstable flavonoid cation backbone. Common anthocyanidins include delphinidin, cyanidin, petunidin, peonidin, malvidin, and pelargonidin, predominantly present in fruits and vegetables like blueberries and tomatoes Khoo et al., 2017. Anthocyanidins exhibit antioxidant properties, primarily through ROS and nitrogen species scavenging, with their activity influenced by the position of hydroxyl and methoxy groups in their structure. Cyanidin-3-glucoside, a natural anthocyanin with antioxidant and anti-inflammatory properties, is metabolized in the gastrointestinal tract to bioactive phenolic metabolites, enhancing its bioavailability and supporting mucosal barrier function and the microbiome (Tan et al., 2019). Cyanidin-3-glucoside has been shown to exert ferroptosis inhibition-related therapeutic effects by activating GPX4-related antioxidant pathways and reducing Fe2+ accumulation in both renal ischemia/reperfusion injury models and myocardial ischemia/reperfusion injury models. (Shan et al., 2021; Du et al., 2023). However, the role of natural Anthocyanidins in ferroptosis in preclinical studies related to CNS diseases still requires further investigation and exploration.

## 6 Discussion and perspectives

Ferroptosis has become a key focus in CNS injury-related diseases. However, clinical trials of ferroptosis inhibitors have yielded inconsistent results, with concerns about side effects and toxicity, challenging clinical translation (Ryan et al., 2023). Iron overload-induced lipid peroxidation and suppression of antioxidant pathways are the primary triggers of ferroptosis in CNS diseases (Wang Y. et al., 2024). The chemical structure of natural flavonoids imparts antioxidant properties, with hydroxyl groups on rings A and B donating electrons to free radicals, forming stable neutral molecules and inhibiting chain reactions (Kongpichitchoke et al., 2015). The conjugated double bonds between rings A/B and C enhance electron absorption and storage, while the oxygen atom on ring C further facilitates electron donation, amplifying their antioxidant effects (Vo et al., 2019). Therefore, investigating the potential of natural flavonoids in inhibiting ferroptosis to mitigate or delay the progression of central nervous system diseases is justified. As anticipated, our findings show that most natural flavonoids activate antioxidant systems, primarily by modulating the Nrf2/GPX4 axis, with some also affecting ferritin or reducing iron accumulation in neurons, thereby further promoting the inhibition of ferroptosis.

Studies on natural flavonoids in acute CNS injuries, including SAH, ICH, IS, CIRI, SCI, and TBI, dominate the research landscape. Only two studies have demonstrated ferroptosis inhibition in preclinical models of NDDs, with quercetin showing efficacy in PD and eriodictyol in AD, while no studies have been conducted in HD (Lin et al., 2022; Li L. et al., 2022). Among the seven subtypes of natural flavonoids, Flavones have been extensively studied for their ability to inhibit ferroptosis in CNS diseases. Among them, Baicalein (including its precursor Baicalin) has been the most widely researched, demonstrating effective ferroptosis inhibition in various CNS injury models, including SAH-EBI, ICH, IS, CIRI, TBI, and TBI-PTE.

Common parameters used to evaluate the potential of flavonoids in inhibiting ferroptosis include GSH levels, oxidized GSH, MDA, free cellular iron levels, ROS, and expression of genes and proteins related to ferroptosis regulation, such as GPX4, HO-1, Nrf2, and FTH1. However, since ferroptosis was formally proposed in 2012 (Dixon et al., 2012), some earlier studies that might have influenced key ferroptosis-related molecules were missed, which is a limitation of this review. Additionally, research on Chinese herbal compound prescriptions inhibiting ferroptosis is ongoing (Shi Y. et al., 2023; Liu J. et al., 2024), but we focused solely on the biological effects of individual natural compounds, excluding combination therapy studies—another limitation. The third limitation is that while most studies on natural flavonoids in animal models have used intraperitoneal injection or oral gavage, the emerging use of nanocarriers and other technologies to enhance bioavailability and targeting has not been emphasized in this review (Khan et al., 2021; Abdi et al., 2024).

Given the lack of clinical trials to validate the efficacy of natural flavonoids in CNS disease patients and the challenges in translating animal data to clinical settings, this study focuses on the application details of natural flavonoids in CNS disease animal models, including administration routes, dosages, and treatment durations. This detailed discussion helps expand *in vivo* study designs, aiming to establish reasonable standards for evaluating biological efficacy and gradually facilitate the progression of clinical trials. In addition to regulating ferroptosis pathways, flavonoids also target multiple genes and signaling pathways, including nuclear receptors, aryl hydrocarbon receptor (AhR), kinases, receptor tyrosine kinases (RTKs), and G protein-coupled receptors (GPCRs) (Safe et al., 2021). This multi-target effect has raised concerns regarding their potential toxicity and drug-drug interactions in clinical applications. To evaluate their therapeutic potential and predict possible side effects, understanding the molecular mechanisms of flavonoids’ actions should be a core focus of future research. Moreover, many flavonoids have relatively low water solubility and bioavailability, making it critical to improve their bioavailability and pharmacokinetics for clinical applications. Techniques such as nanocarriers and crystallization modifications can optimize drug delivery. As our understanding of flavonoids’ role in regulating ferroptosis deepens, research should not only explore the involved molecular pathways but also address the practical aspects of delivering these compounds to specific cells, which is crucial for translating research into effective clinical interventions.

Overall, this study not only investigates the mechanisms of ferroptosis and its interaction with natural flavonoids but also emphasizes the application details of these compounds in preclinical CNS disease models, providing new insights and opportunities for improving CNS disease prognosis and developing neuroprotective agents.

## Glossary

CNS: Central nervous system
SCI: Spinal cord injury
TBI: Traumatic brain injury
IS: Ischemic stroke
CIRI: Cerebral ischemia-reperfusion injury
SAH: Subarachnoid hemorrhage
ICH: Intracerebral hemorrhage
NDDs: Neurodegenerative diseases
AD: Alzheimer’s disease
PD: Parkinson’s disease
HD: Huntington’s disease
ROS: Reactive oxygen species
L-OOHs: Lipid hydroperoxides
**Fe** ^**2+**^: Ferrous iron
LOX: Lipoxygenase
ACSL4: Acyl-CoA synthetase long-chain family member 4
GPX4: Glutathione peroxidase 4
Nrf2: Nuclear factor E2-related factor 2
ESCRT-III: Endosomal sorting complexes required for transport III
PUFAs: Polyunsaturated fatty acid‐phospholipids
PLs: Phospholipids
SFAs: Saturated fatty acids
MUFAs: Monounsaturated fatty acids
PUFA-PLs: Polyunsaturated fatty acid-phospholipids
AA: Arachidonic acid
AdA: Adrenic acid
PEs: Phosphatidylethanolamines
LPCAT3: Lysophosphatidylcholine acyltransferase 3
TZDs: Thiazolidinediones
TRO: Troglitazone
PIO: Pioglitazone
ROSI: Rosiglitazone
•OH: Hydroxyl radicals
LO•: Lipid alkoxyl radicals
L•: Lipid radicals
LOO•: Lipid peroxyl radicals
**H**_**2**_ **O**_**2**_: Hydrogen peroxide
**OH** ^**−**^: Hydroxide ions
COXs: Cyclooxygenases
LB1: LOXBlock-1
4-HNE: 4-hydroxy-2-nonenal
MDA: Malondialdehyde
Hb: Hemoglobin
TF: Transferrin
TfR: Transferrin receptor
FT: Ferritin
FPN: Ferroportin
DMT1: Divalent metal transporter 1
LIP: Labile iron pool
RNAi: RNA interference
FTH1: Ferritin Heavy Chain 1
FTL: Ferritin Light Chain
NCOA4: Nuclear receptor coactivator 4
UPS: Ubiquitin-proteasome system
ATM: Ataxia-telangiectasia-mutated
PROM2: Prominin 2
MVBs: Multivesicular bodies
System Xc-/GSH/GPX4 axis: Cystine/glutamate antiporter system-glutathione-glutathione peroxidase 4 axis
FSP1/CoQ10/NADPH axis: Ferroptosis suppressor protein 1-coenzyme Q10-nicotinamide adenine dinucleotide phosphate axis
GCH1/BH4/DHFR axis: GTP cyclohydrolase 1-tetrahydrobiopterin-dihydrofolate reductase axis
SLC7A11: Light chain subunit solute carrier family 7 member 11
SLC3A2: Heavy chain subunit solute carrier family 3 member 2
GSH: Glutathione
TXNRD1: Thioredoxin reductase 1
GCL: Glutamate-cysteine ligase
GSS: Glutathione synthase
GSR: Glutathione-S reductase
ATF3: Activating transcription factor 3
ATF4: Activating transcription factor 4
mTOR: Mammalian target of rapamycin
mTORC1: MTOR complex 1
Keap1: Kelch-like ECH-associated protein 1
Cul3: Cullin 3
Rbx1: RING-box protein 1
sMAF: Small Maf proteins
ARE: Antioxidant response elements
PPP: Pentose phosphate pathway
G6PD: Glucose-6-phosphate dehydrogenase
HO-1: Heme Oxygenase-1
BBB: Blood-brain barrier
MCAO: Middle cerebral artery occlusion
HIF-1α: Hypoxia-inducible factor-1α
LncRNA: Long non-coding RNA
NF-κB: Nuclear factor kappa-light-chain-enhancer of activated B cells
cPLA2α: Cytosolic phospholipase A2α
NMDA: N-methyl-D-aspartate
pMCAO: Permanent middle cerebral artery occlusion
circRNAs: Circular RNAs
OGD/R: Oxygen-glucose deprivation/reperfusion
MCAO/R: Middle cerebral artery occlusion/reperfusion
NAC: N-acetylcysteine
tMCAO: Transient middle cerebral artery occlusion
EBI: Early brain injury
DBI: Delayed brain injury
ATG5: Autophagy-related gene 5
CSF: Cerebrospinal fluid
SIRT1: Epigenetic regulator Sirtuin 1
CCI: Controlled cortical injury
EAAT1: Excitatory amino acid carrier type 1
Aβ: Amyloid-beta
NFTs: Neurofibrillary tangles
SNpc: Substantia nigra pars compacta
SN: Substantia nigra
DA: Dopaminergic
Htt: Huntingtin
mHtt: Mutant Huntingtin
MRI: Magnetic resonance imaging
QSM: Quantitative susceptibility mapping
3-NP: Hippocampus of 3-nitropropionic acid
DFO: Deferoxamine
DFP: Deferiprone
DFX: Deferasirox
AKI: Acute kidney injury
CPX: Ciclopirox
NSCLC: Non-small cell lung cancer
2,2-BP: 2,2-Bipyridyl
1,10-PT: 1,10-Phenanthroline
RTAs: Radical-trapping antioxidants
GSSH: Glutathione hydropersulfide
Se: Selenium
α-TOH: α-Tocopherol
VK: Vitamin K
VK1: Vitamin K1
RSSH: Hydropersulfide
RSSH/TSP: Hydropersulfides/trans-sulfuration
Fer-1: Ferrostatin-1
Lip-1: Liproxstatin-1
Lip-2: Liproxstatin-2
PTZs: Phenothiazines
PNXs: Phenoxazines
TEMPO: Tetramethylpiperidine-N-oxyl
BHT: Butylated Hydroxytoluene
BHA: Butylated Hydroxyanisole
RPE: Retinal pigment epithelial
D-PUFAs: Deuterated PUFAs
BXM: Bardoxolone methyl
FINs: Class I ferroptosis inducers
PUFA-PEs: Polyunsaturated fatty acid‐phosphatidylethanolamines
PKC: Protein kinase C
DPP4: Dipeptidyl-peptidase-4
SAEs: Serious adverse events
MDS-UPDRS: Movement Disorder Society-Unified Parkinson’s Disease Rating Scale
ADL: Activities of daily living
DAT: Enhanced dopamine transporter
CMRglu: Cerebral metabolic rate of glucose
ADAS-Cog: Alzheimer’s Disease Assessment Scale-Cognitive Subscale
TIA: Transient ischemic attack
BC: Baicalin
BL: Baicalein
PCC-ABI: Preoptic cistern autologous blood injection
tBCCAO: Transient bilateral common carotid artery occlusion
PTGS2: Prostaglandin-endoperoxide synthase 2
**FeCl**_**3**_: Ferric chloride
FAC: Ferric ammonium citrate
PTE: Post-traumatic epilepsy
FTMT: Mitochondrial ferritin
CHY: Chrysin
HIF-1α: Hypoxia-inducible factor 1α
VTX: Vitexin
GAL: Galangin
BCCAO/R: Bilateral common carotid artery occlusion/reperfusion
MWM: Morris water maze
6-OHDA: 6-hydroxydopamine
QCT: Quercetin
Iso: Isoquercetin
ERD: Eriodictyol
PACs: Proanthocyanidins
EGCG: (−)-Epigallocatechin-3-gallate
DHM: Dihydromyricetin
SI: Soybean Isoflavones
CAL: Calycosin
IC II: Icariside II
SY: Safflower Yellow
CY: Carthamin Yellow
AhR: Aryl hydrocarbon receptor
RTKs: Receptor tyrosine kinases
GPCRs: G protein-coupled receptors
